# Antagonistic chromatin regulation of *LHY* homologs by GmASH2R and GmNF-YC4 fine-tunes soybean flowering time for latitudinal adaptation

**DOI:** 10.1016/j.xplc.2026.101917

**Published:** 2026-05-20

**Authors:** Na Zheng, Can Huang, Chengyang Song, Xiaofei Ma, Wenwen Ren, Xiulin Liu, Zhaohan Zhang, Lingyang Feng, Shangwei Zhong, Xing Wang Deng, Xiao Luo

**Affiliations:** 1 Shandong Key Laboratory of Precision Molecular Crop Design and Breeding, Peking University Institute of Advanced Agricultural Sciences, Shandong Laboratory of Advanced Agricultural Sciences in Weifang, Shandong 261325, China; 2 School of Advanced Agricultural Sciences, Peking University, Beijing 100871, China; 3 State Key Laboratory of Protein and Plant Gene Research, Peking–Tsinghua Center for Life Sciences, and School of Life Sciences, Peking University, Beijing 100871, China

**Keywords:** COMPASS-like complex, H3K4 methylation, *GmLHY*s, NF-Y transcription factor, flowering time, photoperiod adaptation

## Abstract

Soybean (*Glycine max*), a photoperiod-sensitive legume critical for global protein and oil supply, relies on precise flowering-time regulation to achieve latitudinal adaptation. *LATE ELONGATED HYPOCOTYL* homologs (*GmLHY*s) promote flowering by repressing the floral inhibitor *E1*; however, the mechanisms that activate *GmLHY*s remain unclear. Although COMPASS (Complex of Proteins Associated with Set1) and COMPASS-like complexes catalyze histone H3 lysine 4 (H3K4) methylation, their functions have diverged considerably across species and remain largely uncharacterized in legumes. Here, we show that GmASH2R1 and GmASH2R2 form a COMPASS-like complex with GmRBL and GmWDR5a proteins that catalyzes genome-wide H3K4 mono-, di-, and trimethylation, revealing roles distinct from those of their counterparts in *Arabidopsis thaliana*. This complex directly activates *GmLHY* expression by promoting H3K4me3 deposition at *GmLHY* promoters, thereby accelerating flowering. Moreover, we uncover a tunable “Yin–Yang” module in which Nuclear Factor Y (NF-Y) subunits GmNF-YC4a and GmNF-YC4b interact with GmASH2R proteins but act as transcriptional repressors. These NF-YC proteins compete for CCAAT-box binding at *GmLHY* promoters, thereby antagonizing GmASH2R-mediated activation and fine-tuning flowering time. Population analyses suggest that the GmASH2R1/2–GmNF-YC4a/b module may have been subject to antagonistic selection during domestication and that specific allelic combinations could contribute to flowering-time plasticity, consistent with adaptive diversification across latitudinal gradients. Our findings establish the COMPASS-like complex as a central activator of flowering in soybean and reveal an epigenetic–transcriptional mechanism that fine-tunes flowering time to support environmental adaptation.

## Teaser

This study reports that a COMPASS-like epigenetic complex containing GmASH2R1 and GmASH2R2 promotes soybean flowering by enhancing H3K4me3 deposition at *GmLHY*s and activating their expression, while two NF-Y transcription factors, GmNF-YC4a and GmNF-YC4b, antagonize this activation, thereby fine-tuning flowering time for latitudinal adaptation.

## Introduction

Flowering time marks a pivotal developmental transition in plants, determining reproductive success and ecological adaptation. In crops, this trait is a major determinant of yield and geographical distribution. Soybean (*Glycine max*), one of the world’s most important crops and a primary source of plant protein and edible oil, is a typical facultative short-day (SD) plant that exhibits pronounced photoperiod sensitivity (Li et al., 2020). This sensitivity not only affects cultivation range and regional adaptability but also determines growth duration and ultimately seed yield (Yu et al., 2023).

Extensive genetic studies have dissected the soybean flowering pathway, revealing a sophisticated regulatory network centered on the legume-specific photoperiod-responsive repressor *E1*, which suppresses the florigen genes *FLOWERING LOCUS T 2a* (*FT2a*) and *FT5a* (Kong et al., 2010; Xia et al., 2012) under non-inductive long days (LDs). Upstream of E1, homologs of the circadian clock components *LATE ELONGATED HYPOCOTYL* (*LHY*) and *CIRCADIAN CLOCK ASSOCIATED 1* (*CCA1*) act as critical promoters of flowering by directly suppressing *E1* transcription. Four soybean *LHY* homologs (*GmLHY1a*, *GmLHY1b*, *GmLHY2a*, and *GmLHY2b*) exert redundant control over flowering time and yield-related traits (Cheng et al., 2019; Dong et al., 2021). Their essential role is underscored by the severe developmental defects observed in the *lhy1a*/*1b*/*2a*/*2b* quadruple mutant, which exhibits extremely delayed flowering and maturity under both LD and SD conditions (Dong et al., 2021), as well as reduced plant height and shortened internodes attributable to diminished endogenous gibberellin levels (Cheng et al., 2019). Notably, *GmLHY1a* (also known as *Tof16*) acts as a central regulator that binds directly to the *E1* promoter, repressing its transcription and thereby relieving repression of *GmFT2a* and *GmFT5a* to promote flowering (Dong et al., 2021). Consistent with the central role of these genes in photoperiodic adaptation, *GmLHY* genes and their regulators, including the evening complex component J and the pseudo-response regulator genes *Tof11* and *Tof12* (orthologs of *Arabidopsis PRR3*), have undergone stepwise selection during soybean domestication and latitudinal expansion (Lu et al., 2020; Dong et al., 2021). Mechanistically, the major long-juvenile trait locus *J* functions by directly binding to the *E1* promoter, thereby repressing its transcription (Lu et al., 2017). Conversely, Tof11 and Tof12 promote adaptation to higher latitudes by directly repressing *GmLHY* expression, which in turn enhances *E1* transcription under LD conditions (Lu et al., 2020). Despite this progress, the upstream regulatory mechanisms governing *GmLHY* expression remain poorly understood, and the mechanisms by which *GmLHY* transcription is activated remain largely unknown.

Transcriptional regulation in eukaryotes is tightly linked to chromatin state, with histone modifications serving as key regulators of developmental transitions and environmental responses in plants (Huang et al., 2025b; Wang et al., 2025c; Xue et al., 2025). Trimethylation of histone H3 lysine 4 (H3K4me3) is a conserved chromatin mark enriched at transcription start sites and strongly associated with transcriptional activation (Luo et al., 2025; Su et al., 2025). Beyond its role in transcriptional initiation, H3K4me3 facilitates RNA polymerase II pause release and elongation (Wang et al., 2023) and antagonizes repressive chromatin states, including DNA methylation (Wang et al., 2025b) and H3K9me3/H3K27me3 modifications (Sankar et al., 2020; Huang et al., 2025b). H3K4 methylation is deposited by evolutionarily conserved COMPASS (COMplex of Proteins Associated with Set1) and COMPASS-like complexes, which were first identified in budding yeast and subsequently characterized in animals and plants (Miller et al., 2001; Takahashi and Shilatifard, 2010). In plants, a core subcomplex comprising ASH2R (*Arabidopsis* Ash2 Relative), RBL (RbBP5-like), and WDR5a is essential for H3K4 methylation. Intriguingly, the role of ASH2R in flowering regulation has diverged substantially among plant species. In *Arabidopsis*, ASH2R primarily maintains H3K4me3 and represses flowering by activating the expression of the flowering repressor *FLOWERING LOCUS C* (*FLC*) (Jiang et al., 2011). By contrast, in rice, ASH2R homologs are required for all three states of H3K4 methylation (H3K4me1/2/3) and activate the key floral integrator *Early Heading Date 1* (*Ehd1*) to promote flowering (Jiang et al., 2018; Zhao et al., 2022). This striking functional divergence of ASH2R, which represses flowering in *Arabidopsis* but promotes flowering in rice, together with its distinct impacts on H3K4 methylation states, underscores the evolutionary plasticity of COMPASS-like complexes and their capacity to integrate into species-specific flowering networks. However, whether COMPASS-like complexes mediate histone methylation to regulate flowering in soybean, and whether *GmLHY* expression is fine-tuned by chromatin modifications during photoperiodic adaptation, remain unknown.

Nuclear Factor Y (NF-Y), a heterotrimeric transcription factor complex composed of NF-YA, NF-YB, and NF-YC subunits, binds to CCAAT-box motifs and modulates chromatin accessibility at target promoters (Wu et al., 2025). In *Arabidopsis*, NF-Y subunits function as central regulators of flowering time, partly by recruiting chromatin modifiers and influencing epigenetic states (Cao et al., 2014; Hou et al., 2014; Huang et al., 2025a). A well-characterized example is the NF-YC3/4/9 complex, which promotes flowering under LD conditions by antagonizing Polycomb-mediated repression and specifically reducing H3K27me3 levels at *FT*, thereby derepressing its expression (Liu et al., 2018; Luo et al., 2018). In soybean, NF-Y complexes likewise regulate key agronomic traits but exhibit functional divergence. For instance, the NF-YA subunit SW14 directly interacts with the NF-YB subunit Leafy Cotyledon1 (GmLEC1) and competitively inhibits the GmLEC1–GmNF-YC2–GmbZIP67 complex to influence seed composition (Zhang et al., 2025). The two soybean homologs of *Arabidopsis NF-YC4* in soybean, *GmNF-YC4a* (*Glyma.06g169600*) and GmNF-YC4b (*Glyma.04g196200*), confer broad-spectrum disease resistance (Qi et al., 2019) and accelerate pod filling and senescence (O'Conner et al., 2021), respectively. Intriguingly, and in contrast to *Arabidopsis*, *GmNF-YC4a* delays flowering and maturation by repressing *GmFT2a*/*5a* under LD conditions (Cai et al., 2024). Despite these advances, the epigenetic mechanisms underlying NF-Y functions in soybean remain poorly understood. Although GmNFYA was reported to compete with the histone deacetylase GmHDA13 for interaction with GmFVE, a homolog of *Arabidopsis* MSI4/FVE to enhance salt tolerance (Lu et al., 2021), it remains unclear whether soybean NF-Y genes coordinate with chromatin-modifying complexes to regulate flowering and whether such interactions have been shaped by selection during soybean domestication.

In this study, we identify a soybean COMPASS-like complex and show that it is antagonized by GmNF-YC4a/b in flowering regulation. We show that two ASH2R homologs, GmASH2R1 and GmASH2R2, act redundantly to promote flowering under both SD and LD conditions and are essential for plant development. GmASH2Rs assemble into a conserved ternary COMPASS-like complex with GmRBL and GmWDR5a, which maintains global H3K4me1/2/3 levels. We further demonstrate that GmASH2Rs directly activate the expression of *GmLHY* genes through H3K4me3 deposition at their promoters, thereby enhancing GmLHY-mediated repression of the floral inhibitor *E1*. Notably, GmNF-YC4a/b physically interact with GmASH2Rs and competitively bind to *GmLHY* promoters via CCAAT-box elements, antagonizing GmASH2R-mediated activation. This establishes a chromatin regulatory module in which GmASH2Rs activate *GmLHY* transcription, while GmNF-YC4a/b provide a counterbalance to fine-tune flowering. Population genomics suggests that *GmASH2R1*/*2* and *GmNF-YC4a*/*b* may have undergone antagonistic selection during domestication, generating latitude-specific allelic combinations that may have contributed to flowering adaptation. Collectively, our findings uncover a domestication-shaped epigenetic–transcriptional mechanism that fine-tunes flowering in soybean.

## Results

### GmASH2R1 and GmASH2R2 act redundantly to accelerate flowering independent of photoperiod

The ASH2R protein is a conserved core subunit of the COMPASS-like complex, which is critical for histone H3K4 methylation and transcriptional regulation. To investigate its function in soybean, we identified two homologs, GmASH2R1 (Glyma.12G020200) and GmASH2R2 (Glyma.11G093700), which share 50% and 49% amino acid identity with *Arabidopsis* ASH2R, respectively (Supplemental Figure 1). Both GmASH2Rs contain a SPRY (SPla and RY anodine receptor) domain, which mediates interaction with RbBP5 and is essential for the assembly and histone methyltransferase activity of COMPASS complexes (Chen et al., 2012). Phylogenetic analysis further revealed that ASH2R proteins from dicots and monocots form two evolutionarily distinct clades with full bootstrap support, separate from the human homolog (Supplemental Figure 2A), supporting the functional conservation of ASH2R within plants.

As core components of the COMPASS-like complex, ASH2R proteins are predicted to function in nuclear chromatin-mediated transcriptional regulation. To confirm this, we determined the subcellular localization of GmASH2R1 and GmASH2R2 by fusing GFP to their N-termini and transiently expressing them in tobacco leaves. Confocal microscopy revealed exclusive nuclear localization of both fusion proteins (Supplemental Figure 2B), consistent with their putative role in chromatin modification. To gain further insight into their biological roles, we analyzed the expression patterns of *GmASH2R1* and *GmASH2R2*. RT-qPCR showed that both genes were constitutively expressed in roots, stems, leaves, flowers, and pods, with maximal levels in flowers (Supplemental Figure 2C). Importantly, transcript levels of both genes were generally upregulated under LD compared with SD conditions across most organs, suggesting that their function may be particularly important under LD photoperiods.

Given previous reports that *ASH2R* plays divergent roles in flowering regulation in *Arabidopsis* and rice, we generated single and double mutants of *GmASH2R1* and *GmASH2R2* in the Williams 82 background using CRISPR–Cas9 (clustered regularly interspaced short palindromic repeats–CRISPR-associated protein 9) genome editing. A 120-bp deletion in *ash2r1-1* is predicted to remove 40 amino acid residues from the ASH2R1 protein, whereas a 1-bp insertion in *ash2r2-1* and a 5-bp deletion in *ash2r2-2* each caused a frameshift mutation (Supplemental Figure 3).

We found that the *ash2r1* and *ash2r2* single mutants flowered at times similar to those of the wild type (WT) under both natural and controlled photoperiod conditions. However, the *ash2r1*/*2* double mutants exhibited severely delayed flowering and dwarf plant architecture in field trials under natural LD conditions in Weifang and natural SD conditions in Sanya (Figures 1A and 1B). This late-flowering and growth-impaired phenotype was confirmed in controlled environments, where two independent double-mutant lines flowered significantly later than the WT under both LD and SD conditions, with the most pronounced delay of more than 20 days observed under LD conditions (Figures 1C–1E). Overexpression of either *GmASH2R1* or *GmASH2R2* alone did not alter flowering time (Supplemental Figure 4). Consistent with the close relationship between flowering time and maturation, the *ash2r1*/*2* double mutants also failed to complete seed development within the normal growing season and produced significantly fewer seeds in both locations (Figures 1A and 1B). Collectively, these results demonstrate that GmASH2R1 and GmASH2R2 act redundantly to promote flowering in a photoperiod-independent manner and are essential for normal plant growth, maturation, and fertility in soybean.

**Figure 1:**
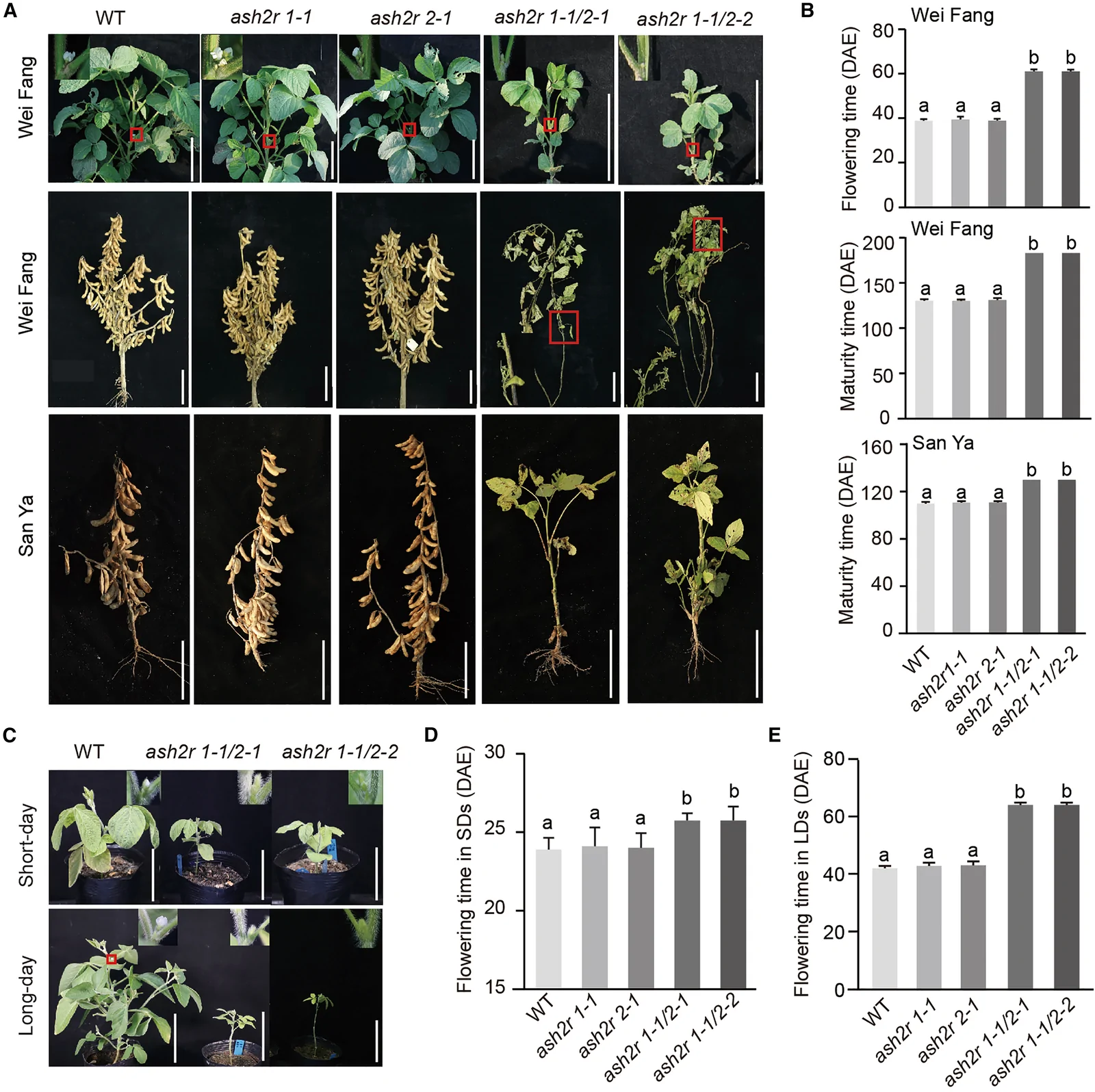
Soybean *ASH2R1* and *ASH2R2* redundantly regulate flowering time and maturation. **(A)** Phenotypes of the WT (Wm82), *ash2r1-1*, *ash2r2-1*, *ash2r1-1*/*2-1*, and *ash2r1-1*/*2-2* mutants grown under natural long-day (LD) conditions in Weifang and natural short-day (LD) conditions in Sanya. **(B)** Flowering and maturation time of the WT and mutants grown in Weifang and Sanya. DAE, days after emergence. Note that the *ash2r1-1*/*2-1* and *ash2r1-1*/*2-2* double mutants had not reached maturity by the end of the observation period; therefore, the values shown represent the final recorded observation days. **(C)** Phenotypes of the WT and mutants grown in a greenhouse under SD and LD conditions. **(D and E)** Flowering time of the WT and mutants grown under SD **(D)** and LD conditions **(E)**. In **(B)**, **(D)**, and **(E)**, different letters indicate statistically significant differences as determined by one-way ANOVA followed by Tukey’s multiple comparisons test. In **(A)** and **(C)**, scale bars represent 10 cm.

### Soybean ASH2R proteins form the core of a conserved COMPASS-like complex with RBL and WDR5a

To determine whether GmASH2R1 and GmASH2R2 are components of a conserved COMPASS-like complex in soybean, we first identified their potential interacting partners. BLAST searches against SoyBase using *Arabidopsis* RBL and WDR5 sequences as queries identified five GmRBL homologs (GmRBL1–GmRBL5) and four GmWDR5 homologs (GmWDR5a1/a2 and GmWDR5b1/b2) (Supplemental Figures 5A and 5B). Phylogenetic analysis showed that plant RBL proteins form a monophyletic clade, with GmRBLs grouping closely with *Arabidopsis* RBL. Similarly, GmWDR5a1/2 clustered with WDR5a from other plants and human WDR5, whereas WDR5b homologs formed a separate clade (Supplemental Figure 5A).

We next investigated whether soybean GmASH2R proteins associate with GmRBLs and GmWDR5a proteins to form the conserved ternary subcomplex required for H3K4 methylation. Subcellular localization analysis revealed that, similar to GmASH2Rs, GmRBL1–3 localized exclusively to the nucleus, whereas GmWDR5a1/2 were present in both the nucleus and cytoplasm (Supplemental Figures 5C and 5D), providing the spatial basis for their interaction in the nucleus. Yeast two-hybrid (Y2H) assays showed that both GmASH2R1 and GmASH2R2 interact directly with GmRBL1/2/3. Notably, these GmRBLs specifically interacted with GmWDR5a1 but not GmWDR5a2, and no direct interaction was detected between the GmASH2Rs and either GmWDR5a protein (Figure 2A and Supplemental Figures 6A and 7). These pairwise interaction patterns suggest that GmRBLs bridge GmASH2R proteins and GmWDR5a1 rather than supporting direct binding between GmASH2Rs and GmWDR5a proteins. The selective interaction of GmRBLs with GmWDR5a1, but not GmWDR5a2, further suggests potential functional divergence among WDR5a paralogs retained after soybean paleopolyploidization. We next tested these interactions *in planta* using bimolecular fluorescence complementation (BiFC). Co-expression of nYFP-GmASH2R1 or nYFP-GmASH2R2 with GmRBL1/2/3-cEYFP reconstituted YFP fluorescence in the nuclei of *Nicotiana benthamiana* cells (Figure 2B and Supplemental Figure 6B). Similarly, nuclear fluorescence was observed when nYFP-GmWDR5a1 was paired with GmRBL1/2/3-cEYFP (Figure 2B and Supplemental Figure 6B). To verify the formation of this ternary complex *in vivo*, we performed co-immunoprecipitation (co-IP) assays in transgenic soybean hairy roots. FLAG-tagged GmRBL1 co-precipitated with both GmASH2R1-HA and GmWDR5a1-HA (Figures 2C and 2D). Together, these results support a model in which GmASH2R, GmRBL, and GmWDR5a form a conserved core subcomplex of the COMPASS-like complex in soybean, with GmASH2R being incorporated into the complex through GmRBL-mediated association.

**Figure 2:**
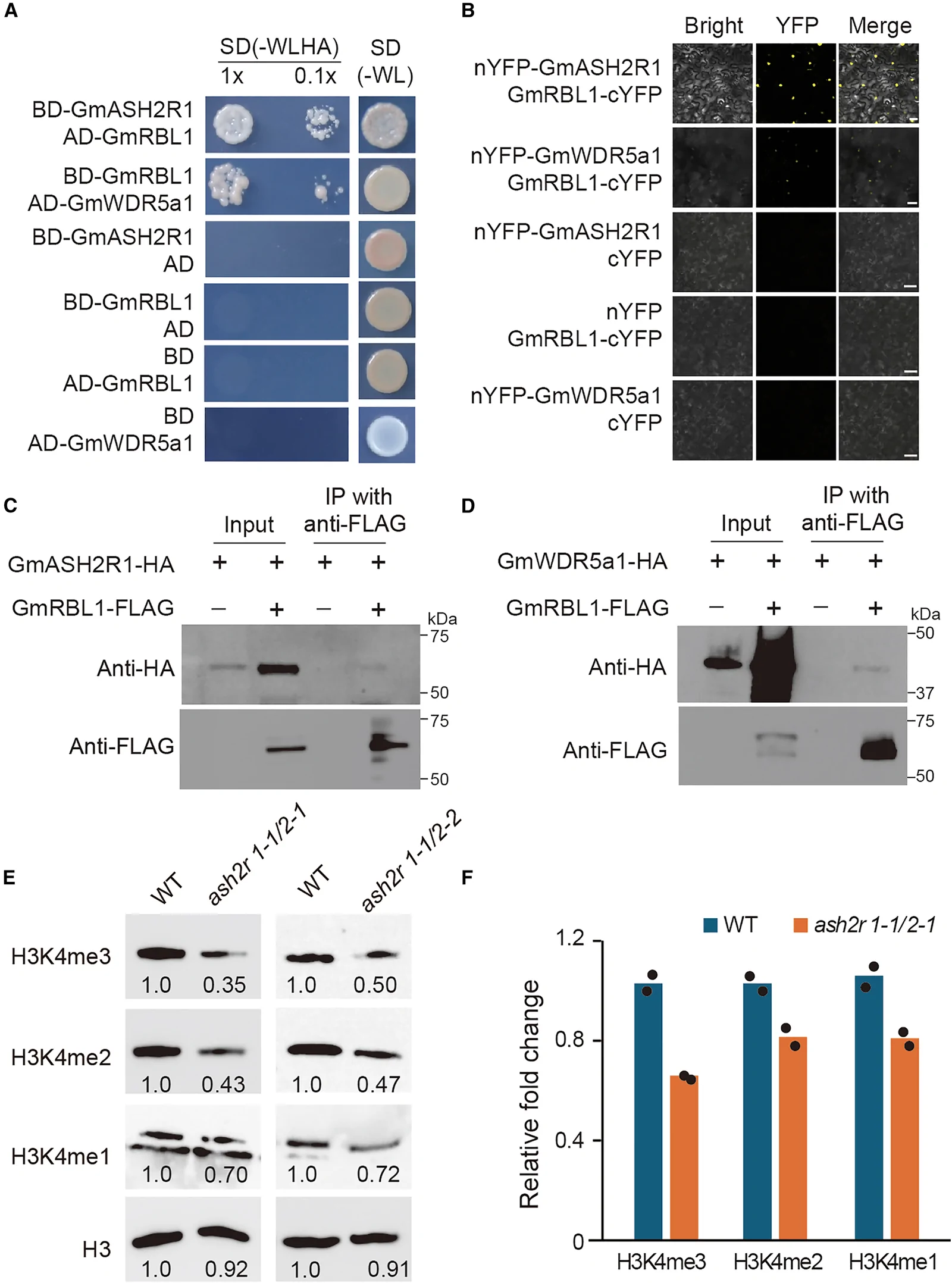
GmASH2R, GmRBL, and GmWDR5a proteins form a nuclear COMPASS-like complex in soybean. **(A)** Yeast two-hybrid assays showing that GmRBL1 interacts with both GmWDR5a1 and GmASH2R1. Full-length GmASH2R1 and GmRBL1 were fused to the GAL4 DNA-binding domain (BD), whereas GmRBL1 and GmWDR5a1 were fused to the GAL4 activation domain (AD). Yeast cells, either undiluted or 10-fold diluted, were spotted onto stringent selective synthetic defined medium lacking tryptophan, leucine, histidine, and adenine (SD/–Trp/–Leu/–His/–Ade). Cells spotted on non-selective synthetic defined medium lacking tryptophan and leucine (SD/–Trp/–Leu) served as growth controls. **(B)** BiFC analysis confirming the nuclear interactions of GmRBL1 with GmWDR5a1 and GmASH2R1 in *N. benthamiana* leaf cells. Scale bars, 50 μm. **(C and D)** Co-immunoprecipitation (co-IP) assays confirming the *in vivo* interactions of GmRBL1-FLAG with GmASH2R1-HA **(C)** and GmWDR5a1-HA **(D)** in soybean hairy roots. Co-IP assays were performed using total proteins extracted from transgenic hairy roots, followed by western blotting. The experiments were performed twice with similar results. **(E and F)** Western blot analysis of H3K4me1, H3K4me2, and H3K4me3 levels in the leaves of WT, *ash2r1-1*/*2-1*, and *ash2r1-1*/*2-2* plants. Total histones were extracted from the youngest leaves of 3-week-old soybean plants. Representative blots are shown in **(E)**. Relative H3K4me1, H3K4me2, and H3K4me3 levels in WT and *ash2r1-1*/*2-1* mutants from two independent biological replicates are shown in **(F)**.

### GmASH2R1 and GmASH2R2 are required for global H3K4 mono-, di-, and trimethylation in soybean

Despite its conserved role as a core component of COMPASS-like complexes, ASH2R’s specific contribution to H3K4 methylation varies among species. In *Arabidopsis*, loss of ASH2R function primarily affects H3K4me3 but not H3K4me1 or H3K4me2 (Jiang et al., 2011), whereas in rice, mutations in ASH2R homologs lead to broad reductions in all three methylation states (Zhao et al., 2022). To determine the role of GmASH2Rs in histone modification, we analyzed global H3K4 methylation levels in the *ash2r1*/*2* double mutants. Total histones were extracted from the leaves of 3-week-old WT and mutant plants harvested at Zeitgeber time 4 (ZT4) under LD conditions. Western blot analysis using modification-specific antibodies revealed that H3K4me1, H3K4me2, and H3K4me3 levels were all markedly reduced in both the *ash2r1-1*/*2-1* and *ash2r1-1*/*2-2* double mutants compared with the WT (Figures 2E and 2F). These results indicate that, unlike in *Arabidopsis* and similar to rice, GmASH2R-containing COMPASS-like complexes are essential for maintaining all three methylation states of H3K4 in soybean.

To characterize the genome-wide transcriptional consequences of GmASH2R loss, we conducted transcriptome sequencing on the leaves of 3-week-old WT and *ash2r1-1*/*2-1* mutants grown under LD conditions. Principal component analysis and correlation analysis confirmed high reproducibility among biological replicates, with one outlier sample (*ash2r1*/*2*_3) excluded from subsequent analyses (Supplemental Figures 8A and 8B). Differential expression analysis identified 6,017 downregulated differentially expressed genes (DEGs) and 7,207 upregulated DEGs in the mutant compared with the WT (Supplemental Figures 8C and 8D). Kyoto Encyclopedia of Genes and Genomes (KEGG) pathway enrichment analysis revealed that downregulated DEGs were predominantly associated with energy production and biosynthetic pathways, including photosynthesis, carbon fixation, and porphyrin metabolism (Supplemental Figure 8E). Thus, the widespread downregulation of genes involved in photosynthetic and biosynthetic metabolism may contribute to the restricted growth and developmental defects observed in the mutant.

To assess the functional relationship between H3K4 methylation states and transcriptomic changes, we integrated RNA sequencing (RNA-seq) data from the *ash2r1*/*2* mutants with genome-wide H3K4 methylation chromatin immunoprecipitation sequencing (ChIP-seq) profiles (Liu et al., 2025). Among the 4,160 H3K4me1/2/3 peaks identified genome-wide, 964 were associated with DEGs (Supplemental Figure 8F). These DEGs exhibited mark-specific associations with expression changes: genes upregulated in *ash2r1*/*2* were significantly enriched in H3K4me2-marked regions, including co-marked H3K4me2/3 peaks (196 genes) and H3K4me2-only peaks (240 genes) (Supplemental Figure 8G). Conversely, downregulated genes were predominantly associated with H3K4me3-specific peaks (100 genes) (Supplemental Figure 8H). Notably, co-marked H3K4me2/3 peaks were found in both up- and downregulated gene categories. These findings indicate that, in soybean, H3K4me3 is generally associated with active transcription, whereas H3K4me2 may mark genes in a poised or repressed state, consistent with studies in other plant species (Liu et al., 2019; Liu et al., 2024; Forestan et al., 2020).

### GmASH2Rs directly promote the expression of *GmLHY*s

Among the DEGs identified by RNA-seq, four core circadian clock homologs, *GmLHY1a*, *GmLHY1b*, *GmLHY2a*, and *GmLHY2b*, were notably downregulated in *ash2r1-1*/*2-1* (Supplemental Figure 8I). This expression pattern was further validated by RT-qPCR (Figure 3A). Given the reported role of the four *GmLHY* genes as redundant regulators of flowering and yield (Dong et al., 2021), with GmLHY1a playing a central role by directly suppressing the key repressor *E1* to permit expression of the florigen genes *GmFT2a* and *GmFT5a* (Lu et al., 2020; Dong et al., 2021), we examined the transcript levels of these flowering pathway genes. In *ash2r1*/*2* double mutants grown under LD conditions, *E1* expression was elevated, whereas the expression of *GmFT2a* and *GmFT5a* were significantly reduced (Figures 3B and 3C). Under SD conditions, most of the *GmLHY* genes were also downregulated, accompanied by decreased *GmFT5a* expression (Supplemental Figures 9A and 9B). These results establish GmASH2R1 and GmASH2R2 as essential regulators of the *GmLHY*-mediated flowering pathway under both LD and SD conditions.

**Figure 3:**
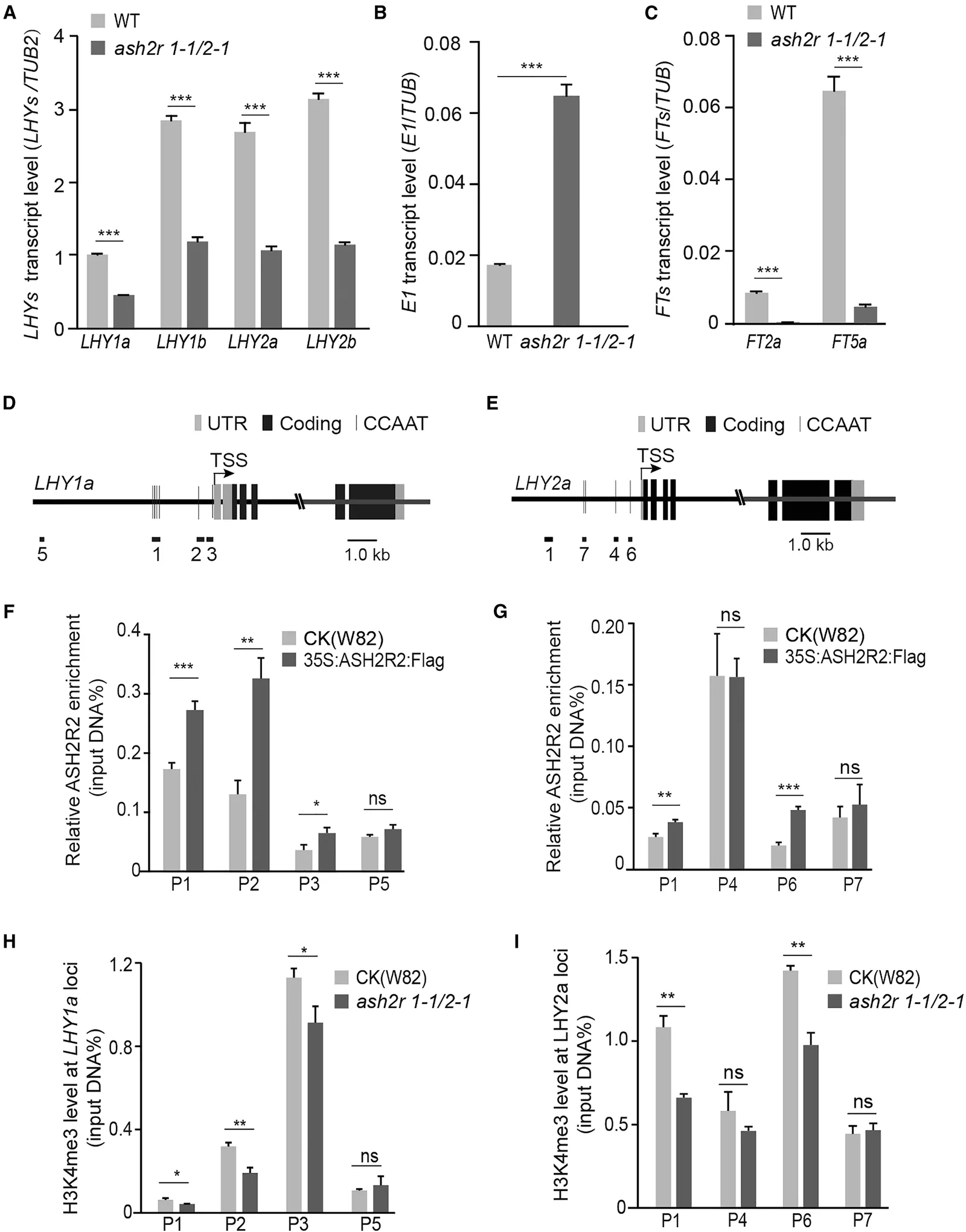
GmASH2Rs activate *GmLHY*s expression through direct chromatin binding and H3K4me3 deposition. **(A–C)** Relative transcript levels of *GmLHY1a*, *GmLHY1b*, *GmLHY2a*, and *GmLHY2b***(A)**; *GmE1***(B)**; and *GmFT2a*/*5a***(C)** in WT and *ash2r1-1*/*2-1* mutants under LD conditions, as quantified by RT-qPCR. Transcript levels were normalized to the endogenous control *Tubulin*. **(D and E)** Schematic structures of *GmLHY1a***(D)** and *GmLHY2a***(E)**. Solid lines indicate the regions examined by ChIP-qPCR. **(F and G)** ChIP-qPCR analysis of GmASH2R2-FLAG enrichment at *GmLHY1a***(F)** and *GmLHY2a***(G)** chromatin in 35Spro:*GmASH2R2-FLAG* transgenic plants. Anti-FLAG-immunoprecipitated genomic fragments were quantified by qPCR and normalized to input DNA. **(H and I)** ChIP-qPCR analysis of H3K4me3 levels at *GmLHY1a***(H)** and *GmLHY2a***(I)** chromatin in WT and *ash2r1-1*/*2-1* mutants. Anti-H3K4me3-immunoprecipitated genomic fragments from each examined region were quantified by qPCR and normalized to input DNA. For **(A–C)** and **(F–I)**, leaves were harvested from 3-week-old seedlings at Zeitgeber time 4 (ZT4) under LD conditions. Data are means ± standard deviation from three independent biological replicates. Asterisks indicate significant differences compared with WT, as determined by two-tailed Student’s *t*-test: ∗∗∗*p* < 0.001, ∗∗*p* < 0.01, ∗*p* < 0.05; ns, not significant.

To determine whether GmASH2Rs directly regulate *GmLHY* genes, we performed ChIP-qPCR using transgenic plants expressing GmASH2R2-FLAG. Significant enrichment was detected at the promoters of *GmLHY1a* and *GmLHY2a*, the genes selected for analysis (Figures 3D–3G), indicating direct binding to these loci. Together, these findings demonstrate that GmASH2Rs regulate flowering in part through direct transcriptional control of *GmLHY*s.

### GmASH2Rs maintain H3K4me3 levels at *GmLHY* chromatin

Given that GmASH2R2 binds to *GmLHY* promoters and that global H3K4me3 levels are reduced in the *ash2r1*/*2* mutant, we asked whether the downregulation of *GmLHY* expression results from diminished H3K4 trimethylation, a chromatin mark associated with active transcription. ChIP-qPCR analysis revealed significantly lower H3K4me3 enrichment at multiple promoter regions of *GmLHY1a* (P1–P3) and *GmLHY2a* (P1 and P6) in *ash2r1*/*2* mutants compared with the WT (Figures 3H and 3I). These regions coincide with GmASH2R binding sites, supporting a model in which GmASH2R binding facilitates H3K4me3 deposition to maintain active transcription of *GmLHY* genes.

### GmNF-YC4a and GmNF-YC4b link GmASH2Rs to *GmLHY* promoters through CCAAT elements

As GmASH2Rs lack a recognizable DNA-binding domain, we sought to identify transcription factors that could mediate their recruitment to *GmLHY* promoters. Using a Y2H screen, we found that GmASH2R2 directly interacts with GmNF-YC4a and GmNF-YC4b (Figure 4A). We confirmed this interaction *in planta* through a luciferase complementation imaging assay (Figures 4B and 4C) and co-IP experiments in *N. benthamiana* leaves co-expressing GmASH2R2-FLAG and GmNF-YC4a/4b-HA (Figures 4D and 4E). Together, these results indicate that GmASH2R2 physically associates with both GmNF-YC4a and GmNF-YC4b.

**Figure 4:**
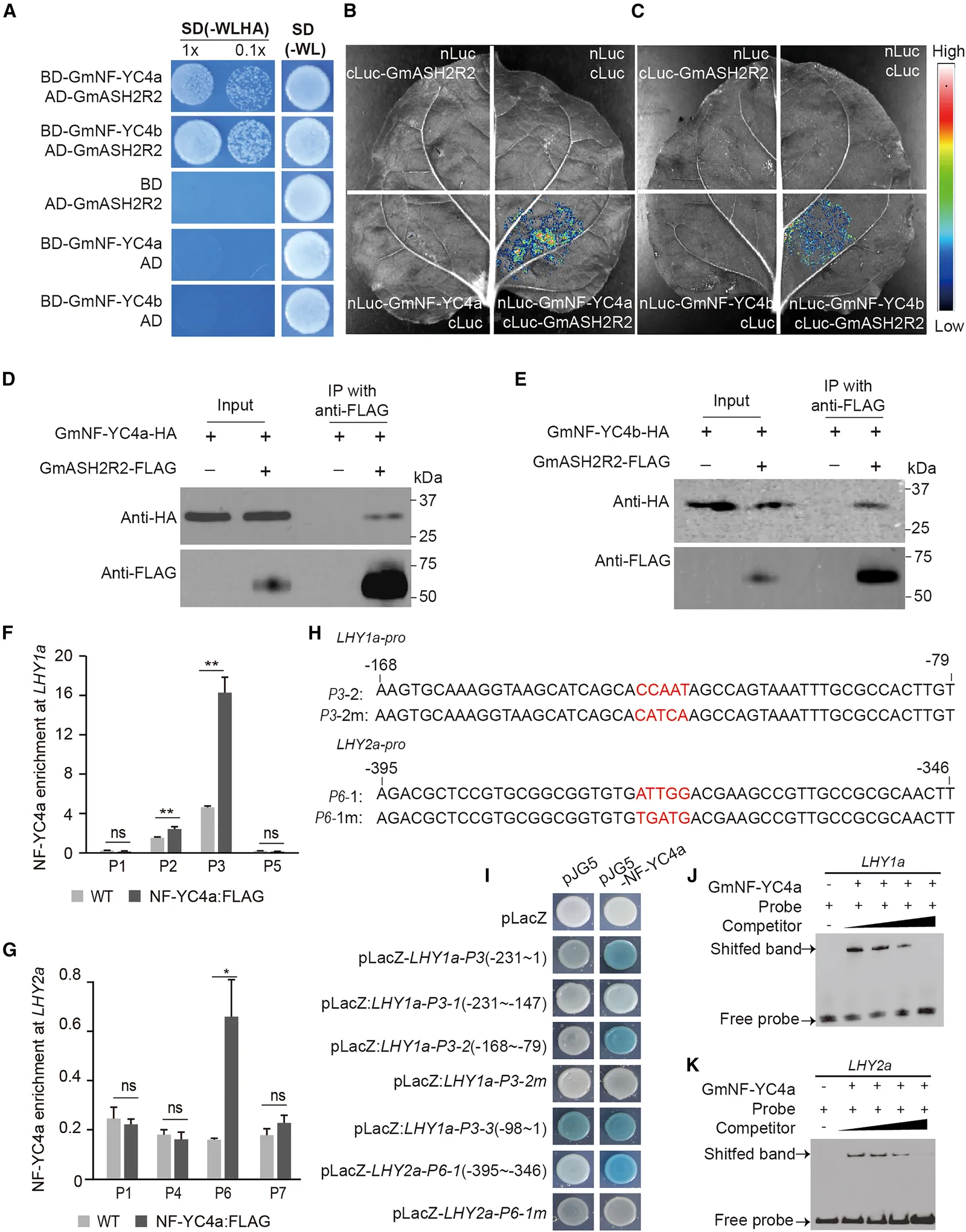
GmNF-YC4a/4b directly bind CCAAT motifs in *GmLHY1a* and *GmLHY2a* promoters and interact with GmASH2R2. **(A)** Y2H assays demonstrating interactions between GmASH2R2, fused to the GAL4 activation domain (AD), and GmNF-YC4a or GmNF-YC4b, fused to the GAL4 DNA-binding domain (BD). Yeast cells, either undiluted or 10-fold diluted, were spotted onto synthetic defined medium lacking leucine and tryptophan (SD/−Leu/−Trp) or onto more stringent medium additionally lacking histidine and adenine (SD/−Leu/−Trp/−His/−Ade). **(B and C)** Luciferase complementation imaging assays confirming interactions between GmASH2R2 and GmNF-YC4a **(B)** or GmNF-YC4b **(C)** in *N. benthamiana* leaves. GmASH2R2 was fused to the C-terminal portion of luciferase (cLUC), and GmNF-YC4a or GmNF-YC4b was fused to the N-terminal portion of luciferase (nLUC). The indicated plasmid pairs were transiently co-expressed via *Agrobacterium*-mediated infiltration. Luciferase activity was imaged at 48 h post-infiltration. **(D and E)** Co-IP assays confirming the *in vivo* interactions of GmASH2R2-FLAG with GmNF-YC4a-HA **(D)** and GmNF-YC4b-HA **(E)** in *N. benthamiana* leaves. Total proteins were extracted from transiently transformed leaves and immunoprecipitated with an anti-FLAG antibody, followed by western blotting. The experiments were performed twice with similar results. **(F and G)** ChIP-qPCR analysis of GmNF-YC4a-FLAG enrichment at *GmLHY1a***(F)** and *GmLHY2a***(G)** chromatin. Anti-FLAG-immunoprecipitated genomic fragments were quantified by qPCR and normalized to input DNA. Leaves were harvested from 3-week-old seedlings at ZT4 under LD conditions. Data are means ± standard deviation from three independent biological replicates. Asterisks indicate significant differences compared with WT, as determined by two-tailed Student’s *t*-test: ∗∗*p* < 0.01, ∗*p* < 0.05; ns, not significant. **(H)** Sequences of the *GmLHY1a* and *GmLHY2a* promoter regions used for the Y1H assay and EMSA. Mutated CCAAT motifs are indicated in red. Numbers indicate positions relative to the transcription start site. **(I)** Y1H assays showing that GmNF-YC4a binds to the *GmLHY1a* and *GmLHY2a* promoters via the CCAAT motif. WT, truncated, and CCAAT-mutated promoter fragments fused to LacZ were used as bait. *GmNF-YC4a* cloned into *pJG4-*5 served as the prey construct, and the empty vector served as the control. Yeast cells were cultured on selective SC medium lacking tryptophan and uracil (SC/−Trp/−Ura) and supplemented with galactose, raffinose, and X-Gal. **(J and K)** EMSA showing that GmNF-YC4a binds to the *GmLHY1a***(J)** and *GmLHY2a***(K)** promoter regions via the CCAAT motif. Biotinylated WT probes were incubated in the absence (−) or presence (+) of purified His-GmNF-YC4a protein. Unlabeled WT or mutant probes were added at 1-, 2-, 10-, and 50-fold molar excess relative to the biotinylated probe as competitors. Arrows indicate specific GmNF-YC4a–probe complexes and free probes.

NF-Y transcription factors are classically known to recognize CCAAT-box motifs in eukaryotic promoters (Dolfini et al., 2009). Emerging evidence further indicates that plant NF-YC subunits have acquired intrinsic DNA-binding activity beyond their canonical role within the NF-Y complex (Chaves-Sanjuan et al., 2021). Indeed, ZmNF-YC1 directly binds the ZmAPRG promoter in yeast one-hybrid (Y1H) assays (Bai et al., 2024), and OsNF-YC8/9/10 directly target the SBE3 promoter to promote amylopectin biosynthesis in rice (Chen et al., 2025). These findings establish that plant NF-YC family members can function as sequence-specific DNA-binding proteins. We therefore tested whether GmNF-YC4a binds to *GmLHY* promoters in regions overlapping GmASH2R2 occupancy. ChIP-qPCR using *GmNF-YC4a-FLAG* overexpression plants showed enrichment at the P2 and P3 regions of the *GmLHY1a* promoter and the P6 region of the *GmLHY2a* promoter (Figures 4F and 4G), which coincide with GmASH2R2 binding sites and contain CCAAT motifs (Figures 3D and 3E). Y1H assays further demonstrated that GmNF-YC4a binds directly to specific fragments of the *GmLHY1a* promoter from −168 to −79 bp and the *GmLHY2a* promoter from −395 to −346 bp. Mutation of the CCAAT motif abolished this binding, confirming its necessity for GmNF-YC4a recruitment (Figures 4H and 4I). Furthermore, electrophoretic mobility shift assays (EMSAs) verified this interaction *in vitro*. A clear gel shift was observed upon incubation with GmNF-YC4a, and this shift was abolished by inclusion of a specific unlabeled competitor (Figures 4J and 4K).

Collectively, our results demonstrate that GmNF-YC4a binds to CCAAT-box elements within the *GmLHY* promoters and interacts with GmASH2R-containing COMPASS-like complexes.

### GmNF-YC4a and GmNF-YC4b function redundantly to repress *GmLHY* transcription and delay flowering

Having established that GmNF-YC4a binds to CCAAT-box elements within the *GmLHY* promoters, we next sought to determine its role in transcriptional regulation. We measured the activity of the *GmLHY1a* and *GmLHY2a* promoters fused to firefly luciferase (LUC) in *N. benthamiana leaves using a* firefly *luciferase transient expression assay*. GmNF-YC4a or GmNF-YC4b expression suppressed the LUC activity driven by each promoter, and this repression was further enhanced when the two proteins were co-expressed (Figures 5A–5C). These results demonstrate that GmNF-YC4a and GmNF-YC4b function redundantly as repressors of *GmLHY* transcription.

**Figure 5:**
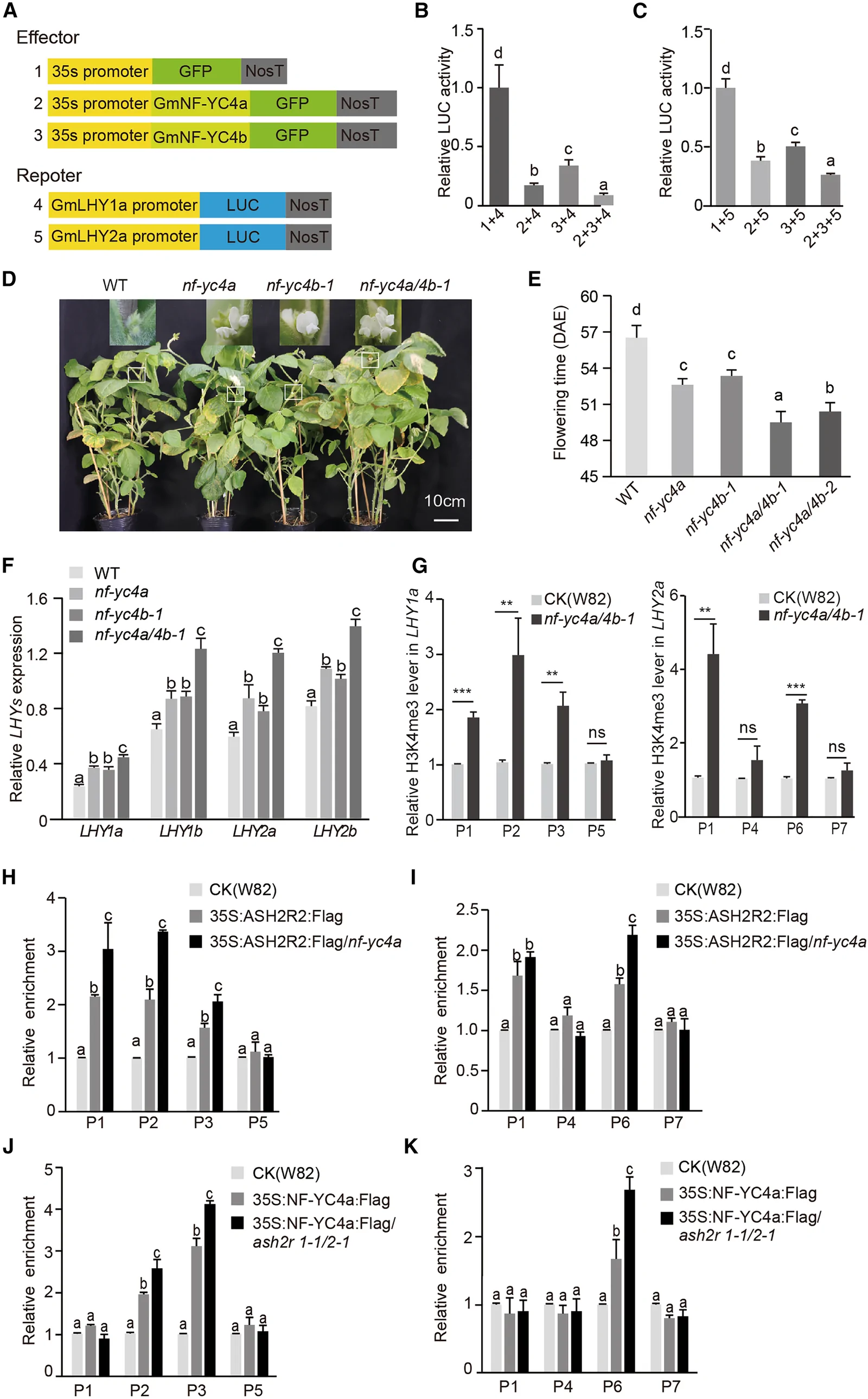
GmNF-YC4a/b repress *GmLHY* expression and antagonize GmASH2R2 binding under long-day (LD) conditions. **(A)** Schematic representation of the constructs used in transient expression assays. 35S, CaMV 35S promoter; LUC, firefly luciferase; NosT, nopaline synthase terminator. **(B and C)** GmNF-YC4a and GmNF-YC4b suppress *GmLHY1a***(B)** and *GmLHY2a***(C)** promoter activity in *N. benthamiana* leaves. **(D and E)** Flowering phenotypes **(D)** and flowering time **(E)** of WT, *nf-yc4a*, *nf-yc4b-1*, and *nf-yc4a*/*4b* double mutants (*nf-yc4a*/*4b-1* and *nf-yc4a*/*4b-2*) grown under LD conditions. Scale bar, 10 cm. Flowering time was recorded as days after emergence (DAE). **(F)** Expression levels of *GmLHYs* in WT and *nf-yc4*a/b mutants. Leaf samples were harvested from 3-week-old seedlings at ZT4 under LD conditions. Transcript levels were quantified by RT-qPCR and normalized to *Tubulin*. **(G)** ChIP-qPCR analysis of H3K4me3 enrichment at the *GmLHY1a* and *GmLHY2a* loci in *nf-yc4a*/*4b-1* double mutants. Leaves were harvested from 3-week-old seedlings at ZT4 under LD conditions. **(H and I)** ChIP-qPCR analysis of GmASH2R2-FLAG enrichment at *GmLHY1a***(H)** and *GmLHY2a***(I)** loci in WT (Wm82) or the *nf-yc4a* background. GmASH2R2-FLAG was transiently expressed in soybean leaves by *Agrobacterium*-mediated infiltration. **(J and K)** ChIP-qPCR analysis of GmNF-YC4a-FLAG enrichment at *GmLHY1a***(J)** and *GmLHY2a***(K)** loci in WT (W82) or *ash2r1-1*/*2-1* double mutant backgrounds. GmNF-YC4a-FLAG was transiently expressed in soybean leaves by *Agrobacterium*-mediated infiltration. For **(G)**–**(K)**, levels of anti-H3K4me3- or anti-FLAG-immunoprecipitated chromatin fragments were quantified by qPCR and normalized to the internal control *Tubulin*. Relative fold-enrichment values were calculated as the ratio of enrichment in H3K4me3-, GmASH2R2-FLAG-, or GmNF-YC4a-FLAG-expressing samples relative to the background control (WT). For **(B)**, **(C)**, and **(G)**–**(K)**, data represent means ± standard deviation of three biological replicates. Different letters indicate statistically significant differences (one-way ANOVA followed by Tukey’s multiple-comparisons test, *p* < 0.05). Asterisks indicate significant differences compared with WT (two-tailed Student’s *t*-test): ∗∗∗*p* < 0.001, ∗∗*p* < 0.01, and ns, not significant.

To investigate the biological function of GmNF-YC4a and GmNF-YC4b in regulating flowering time, we generated *GmNF-YC4a and GmNF-YC4b* single and double mutants using the CRISPR-Cas9 system in the Williams 82 background. Sequencing analysis confirmed that the nf-yc4a allele carried a 3-bp deletion at the sgRNA3 target site and a 4-bp deletion at the sgRNA4 target site, whereas nf-yc4b-1 and nf-yc4b-2 harbored a 2-bp deletion at the sgRNA4 target site and a 1-bp deletion at the sgRNA1 target site, respectively (Supplemental Figure 10). These mutations are predicted to cause frameshifts. Under LD conditions, both the *nf-yc4a* and *nf-yc4b* single mutants flowered significantly earlier than the WT, by approximately 3 days. The *nf-yc4a*/*4b* double mutants showed an even more pronounced early-flowering phenotype, flowering approximately 6 days earlier than the WT and significantly earlier than either single mutant (Figures 5D and 5E). These genetic data demonstrate that *GmNF-YC4a* and *GmNF-YC4b* repress flowering in a partially redundant manner.

We next asked whether the early flowering phenotype of the *nf-yc4a*/*4b* mutants is associated with altered *GmLHY* expression and H3K4me3 enrichment. RT-qPCR analysis of leaf samples collected at ZT4 under LD conditions showed that the transcript levels of all four *GmLHY* genes were significantly elevated in both single mutants compared with the WT (Figure 5F). In addition, ChIP-qPCR analysis revealed significantly higher H3K4me3 enrichment at multiple regions in the *GmLHY1a* (P1–P3) and *GmLHY2a* (P1 and P6) promoters in *nf-yc4a*/*4b-1* mutants compared with the WT (Figure 5G). Moreover, the double mutants showed even stronger upregulation of these genes, consistent with the early flowering phenotype (Figures 5D–5G). These findings reinforce the idea that GmNF-YC4a and GmNF-YC4b function redundantly to repress *GmLHY* transcription and limit H3K4me3 accumulation at *GmLHY* promoters, ultimately delaying flowering.

### GmASH2R2 and GmNF-YC4a compete for binding at *GmLHY* promoters

To further elucidate the regulatory interplay between GmASH2Rs and GmNF-YC4a at *GmLHY* loci, we fused the *GmLHY1a* and *GmLHY2a* promoters to the *LUC* reporter gene and performed dual-luciferase transient expression assays. When co-expressed with GmNF-YC4a, both promoters showed significantly reduced activity (Supplemental Figures 11A and 11C). Notably, this repression was partially alleviated by simultaneous expression of GmASH2R2 (Supplemental Figures 11A and 11C), suggesting that GmASH2R2 counteracts GmNF-YC4a-mediated transcriptional repression.

To determine whether this functional antagonism reflects competitive chromatin binding, we performed ChIP-qPCR assays to monitor promoter occupancy by each factor. We transiently expressed GmASH2R2-FLAG in both WT and *nf-yc4a* plants and assessed its enrichment at *GmLHY* loci. In the absence of *GmNF-YC4a*, GmASH2R2 occupancy at *GmLHY1a* and *GmLHY2a* chromatin was significantly increased (Figures 5H and 5I and Supplemental Figure 12B). Reciprocally, GmNF-YC4a-FLAG enrichment at both promoters was increased in *ash2r1*/*2* mutants compared with the WT (Figures 5J and 5K and Supplemental Figure 12C). These reciprocal changes in chromatin occupancy, combined with the opposing effects of the two factors on promoter activity, demonstrate that GmNF-YC4a and GmASH2R2 compete for binding at *GmLHY* chromatin. This antagonistic relationship provides a mechanism for fine-tuning *GmLHY* expression and consequently modulating flowering time in soybean.

### Stepwise and sustained selection on *GmASH2R1* and *GmASH2R2* drives soybean latitudinal adaptation

Building on the established role of GmASH2Rs in flowering regulation, we next investigated whether these loci experienced selection during soybean domestication and improvement. Population genomic analysis of 4,414 accessions revealed contrasting evolutionary trajectories. *GmASH2R1* showed evidence of sustained selection, with selection pressure acting over an extended period, rather than through a short-lived, instantaneous sweep, enabling the maintenance and gradual enrichment of the corresponding alleles in the population. This pattern was reflected by high differentiation during both domestication and improvement (fixation index [*F*_*ST*_] = 0.178 and 0.164, respectively) and a bottleneck–recovery pattern of nucleotide diversity, with a sharp decline during domestication (π = 0.00330 to 0.00158) followed by partial recovery in cultivars (π = 0.00196), suggesting initial selective sweeps followed by allele introgression (Figure 6A). In contrast, G*mASH2R2* underwent stepwise selection, in which selection effects accumulated in a stepwise manner, followed by continued selection in subsequent stages. This was evidenced by strong wild–landrace differentiation (*F*_*ST*_ = 0.293) that weakened during improvement (*F*_*ST*_ = 0.063), accompanied by a progressive decline in nucleotide diversity (π_wild_ = 0.00187; π_landrace_ = 0.00130; π_cultivar_ = 0.00092), consistent with consecutive selective sweeps (Figure 6B). These distinct selection modes identify both loci as key targets of artificial selection during soybean evolution.

**Figure 6:**
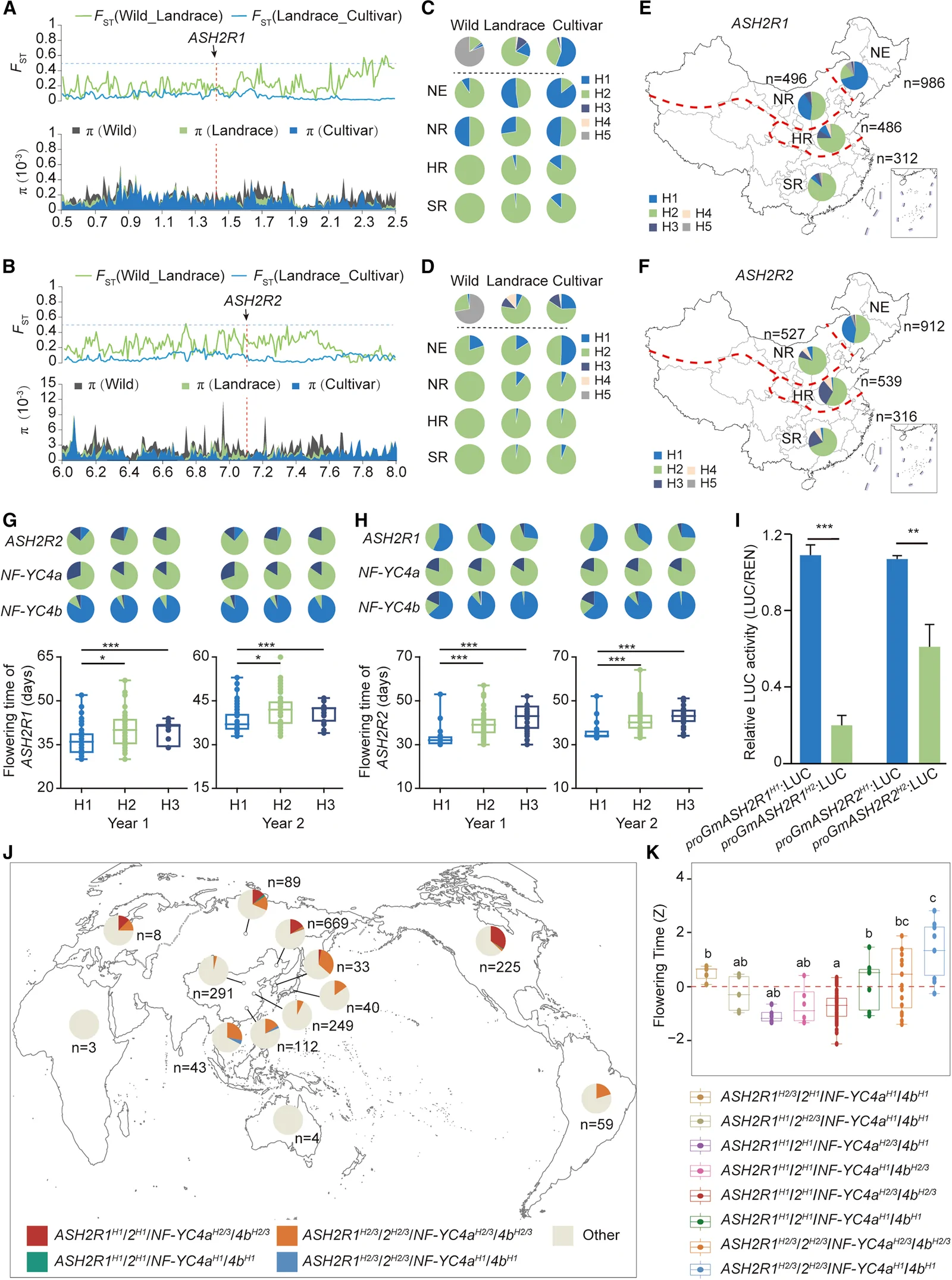
Putative antagonistic selection contributes to the divergence of *GmASH2R1* and *GmASH2R2* and to flowering-time adaptation across soybean geographic regions. **(A and B)** Population differentiation index (*F*_*ST*_) and nucleotide diversity (*π*) values within 2-Mb genomic regions centered on *GmASH2R1***(A)** and *GmASH2R2***(B)** in wild soybean accessions, landraces, and improved cultivars. Blue dashed lines indicate genome-wide thresholds. **(C and D)** Allele frequency distributions of *GmASH2R1***(C)** and *GmASH2R2***(D)** across soybean subpopulations, with the geographic origins of accessions carrying the H1 and H2 alleles indicated. H1 and H2 represent haplotypes, defined as combinations of alleles at linked loci inherited together on the same chromosome; NE, Northeast; NR, North; HR, Huang-Huai; SR, South. **(E and F)** Geographic distributions of *GmASH2R1***(E)** and *GmASH2R2***(F)** alleles across major ecological regions in China. **(G and H)** Flowering time of accessions carrying different *GmASH2R1***(G)** and *GmASH2R2***(H)** alleles in field trials conducted over 2 consecutive years in Henan Province. Pie charts above the boxplots show the relative frequencies of *GmASH2R1*/*2* alleles and their antagonistic partners, *GmNF-YC4a*/*b*, in the tested accessions. Boxplots display the median (center line), interquartile range (box boundaries, Q1–Q3), and whiskers extending to the minimum and maximum values. Individual data points are overlaid. The pie-chart colors correspond to those of the boxplots: H1, blue; H2, light green; and H3, dark blue. The pie charts show the allele frequencies of the indicated background genotypes within the populations represented by the boxplots (H1–H3). **(I)** Relative luciferase activities driven by the promoters of the major H1 and H2 haplotypes of *GmASH2R1* and *GmASH2R2*. The relative LUC/REN ratio was calculated by normalizing firefly luciferase (LUC) activity to *Renilla* luciferase (REN) activity. Data are presented as means ± standard deviation from three independent biological replicates. **(J)** Global distribution of soybean accessions carrying diverse allelic variants of *GmASH2R1*/*2* and *GmNF-YC4a*/*b*. Pie charts indicate the relative frequency of each genotype at the corresponding geographic location. The number of accessions (*n*) is shown for each region. H1/H2, as described in **(C)**. **(K)** Flowering time variation among soybean accessions with different *GmASH2R1*/*2* and *GmNF-YC4a*/*b* haplotypes. Flowering time data for each accession were *Z*-score normalized across multiple environments and years to account for environmental variation. Mean *Z* scores reflect the flowering tendency of each genotype, with negative values indicating earlier flowering and positive values indicating later flowering. Boxplots show the median (center line), interquartile range (box), and distribution range (whiskers). For **(G)–(I)**, statistical significance was determined by two-tailed *t*-tests: ∗*p* < 0.05, ∗∗*p* < 0.01, and ∗∗∗*p* < 0.001. For **(K)**, different letters above the boxplots denote statistically significant differences among genotypes (Welch one-way ANOVA followed by the Games–Howell *post hoc* test, *p* < 0.05). Overall, putative coordinated, antagonistic, and photoperiod-dependent selection on *GmASH2R1*/*2* and *GmNF-YC4a*/*b* may contribute to the latitudinal clines observed in haplotype frequencies, which may thereby fine-tune flowering-time adaptation in soybean.

To determine whether these distinct selection patterns shaped functional allelic diversity, we analyzed sequence variation in the coding regions and 2.5-kb upstream regulatory regions of both genes. For *GmASH2R1*, the wild-enriched haplotype H5 represented the ancestral state, whereas the domestication-enriched haplotypes H1 and H2 showed complementary latitudinal distributions: H1 predominated at high latitudes, whereas H2 was fixed in middle- to low-latitude regions (Figures 6C and 6E, and Supplemental Figures 13A–13C). *GmASH2R2* showed a similar pattern, with ancestral H5 being completely eliminated during domestication and derived H1 and H2 exhibiting the same latitudinal partitioning (Figures 6D and 6F, and Supplemental Figures 13D–13F). These convergent latitudinal clines across the two *GmASH2R* paralogs suggest that artificial selection may have independently modulated GmASH2R-mediated flowering regulation, thereby facilitating the ecological adaptation and geographic expansion of soybean.

### Antagonistic latitudinal selection acting on the GmASH2R1/2–GmNF-YC4a/b module fine-tunes soybean flowering-time adaptation

Given that GmASH2R1/2 and GmNF-YC4a/b competitively regulate *GmLHY* expression and flowering time, we asked whether this regulatory antagonism contributes to geographic adaptation. Population genetic analyses revealed complementary latitudinal clines in *GmASH2R1*/*2* and *GmNF-YC4a*/*b* allele frequencies, consistent with their opposing roles in flowering control (Figures 6E–6H and Supplemental Figures 13G, 13H, 14A, and 14B). The early-flowering alleles *GmASH2R1*^*H1*^ and *GmASH2R2*^*H1*^ predominated in high-latitude accessions (70.6% and 52.5% in northeastern populations, respectively) but declined progressively toward southern latitudes, where the late-flowering alleles *GmASH2R1*^*H2*^ and *GmASH2R2*^*H2*/*H3*^ prevailed (Figures 6E–6H). Conversely, *GmNF-YC4a*/*b* alleles showed the opposite geographic pattern. The late-flowering allele *GmNF-YC4b*^*H1*^ was enriched at low latitudes, reaching 83.6% and 78.3% in Huang-Huai and southern regions, respectively, whereas early-flowering alleles predominated in northern regions (Supplemental Figures 14A and 14B).

This mirror-image distribution reflects photoperiod-dependent adaptive strategies: GmASH2R promotes flowering under both LD and SD conditions, whereas GmNF-YC4 proteins primarily represses flowering under LDs. Consequently, northern accessions may accelerate flowering under extended photoperiods by exploiting GmASH2R-mediated flowering promotion together with attenuated *GmNF-YC4* repression. By contrast, southern accessions may delay flowering under naturally shorter day lengths through reduced GmASH2R activity, rather than relying solely on *GmNF-YC4* repression, which appears to contribute minimally under these conditions. Phenotypic analyses over two growing seasons confirmed these complementary selective patterns. Progressive decreases in the frequencies of the *GmASH2R1*^*H1*^ and *GmASH2R2*^*H1*^ alleles, coupled with increased frequencies of *GmNF-YC4a*^*H1*^ and *GmNF-YC4b*^*H1*^ , were associated with later flowering (Figures 6G and 6H), whereas the opposite allelic shifts were associated with earlier flowering (Supplemental Figures 14A and 14B). We further performed dual-luciferase reporter assays to assess the transcriptional activity of promoters from different haplotypes. The H1 promoter haplotypes of both *GmASH2R1* and *GmASH2R2* exhibited significantly higher transcriptional activity than their respective H2 counterparts (Figure 6I). These findings support a model in which antagonistic latitudinal selection on this regulatory module fine-tunes flowering-time adaptation in soybean.

### Combined allelic variation in *GmASH2R1/2* and *GmNF-YC4a/b* drives global latitudinal adaptation in soybean

To evaluate how antagonistic selection between *GmASH2R1*/*2* and *GmNF-YC4a*/*b* shaped global adaptation, we analyzed worldwide germplasm distributions (Figure 6J). Accessions carrying *GmASH2R1*^*H1*^ and *GmASH2R2*^*H1*^ together with early-flowering GmNF-YC4a/b alleles (designated *GmNF-YC4a*^*H2*/*3*^ and *GmNF-YC4b*^*H2*/*3*^) were restricted to high-latitude regions, including northeast China, Europe, North America, and Russia, whereas those combining late-flowering *GmASH2R1*^*H2*/*3*^ and *GmASH2R2*^*H2*/*3*^ alleles with *GmNF-YC4a*^*H1*^ and *GmNF-YC4b*^*H1*^ were restricted to low-latitude regions, including Southeast Asia and southern China (Figure 6J).

To accurately assess how these allelic combinations influence flowering time, we minimized potential confounding effects from accessions carrying variants in other flowering-related genes (*E3*, *E4*, *Tof18*, *GmFT2a*, and *GmFT5a*) or established loss-of-function alleles (*e1-as*, *J*, *Tof11*, and *Tof12*). *Z*-normalized flowering-time data across multiple environments confirmed that, among high-latitude-adapted accessions carrying *GmASH2R1*^*H1*^ and *GmASH2R2*^*H1*^, those carrying *GmNF-YC4a*^*H2*/*3*^ and *GmNF-YC4b*^*H2*/*3*^ flowered earlier than those carrying *GmNF-YC4a*^*H1*^ and *GmNF-YC4b*^*H1*^ (Figure 6K). Conversely, flowering was further delayed in low-latitude-adapted accessions carrying *GmASH2R1*^*H2*/*3*^ and *GmASH2R2*^*H2*/*3*^ (Figure 6K).

These findings demonstrate that regional selection may have favored specific allelic combinations adapted to local photoperiodic regimes. Given their distinct photoperiod dependencies, northern accessions may exploit early-flowering allelic combinations to accelerate flowering under extended photoperiods, whereas southern accessions appear to utilize late-flowering combinations, particularly attenuated GmASH2R activity, to delay flowering under shorter day lengths. This antagonistic coordination likely contributes to the precise fine-tuning of photoperiodic responses, consistent with soybean domestication and geographic expansion across diverse latitudinal environments.

## Discussion

In this study, we identify a core epigenetic–transcriptional module that fine-tunes flowering time in soybean. The COMPASS-like complex, composed of GmASH2R1/2, GmRBL, and GmWDR5a, maintains global H3K4 mono-, di-, and trimethylation and promotes flowering by directly activating *GmLHY* genes. Remarkably, GmNF-YC4a/b interact with this complex and bind to *GmLHY* promoters via CCAAT-box elements but act as transcriptional repressors that antagonize GmASH2R-dependent activation. This competitive interaction establishes a dynamic chromatin regulatory circuit that balances activation and repression to ensure proper flowering-time control. Population and evolutionary analyses further indicate that components of this antagonistic module may have undergone selection during soybean domestication, potentially contributing to the establishment of latitude-specific allelic combinations that optimize flowering time and facilitate latitudinal adaptation in soybean.

### A conserved COMPASS-like core complex in soybean with divergent roles in H3K4 methylation

The assembly of a ternary ASH2R–RBL–WDR5a subcomplex has been shown to be essential for H3K4 methyltransferase activity in several plant species (Jiang et al., 2011; Zhao et al., 2022; Zhang et al., 2024), although its conservation varies across species (Xu et al., 2024). In soybean, we identified multiple homologs of each subunit and confirmed that GmRBL bridges GmASH2R and GmWDR5a to form a stable nuclear complex. This finding supports the conservation of the COMPASS-like core machinery in legumes. However, despite this structural conservation, the complex exhibits divergent roles in H3K4 methylation across plant species. In contrast to *Arabidopsis*, where ASH2R specifically maintains H3K4me3 (Jiang et al., 2011), GmASH2R is essential for genome-wide H3K4me1/2/3, a functional profile that more closely resembles that of rice than that of *Arabidopsis,* despite soybean and Arabidopsis both being dicots (Jiang et al., 2018; Zhao et al., 2022). These findings suggest that the regulatory scope of ASH2R has undergone lineage-specific diversification, possibly reflecting adaptations related to growth habit, reproductive strategy, or environmental responsiveness. The broader role of GmASH2R across H3K4 methylation states may be linked to soybean’s strong photoperiod sensitivity and the need for coordinated regulation of numerous developmental and metabolic genes.

Integration of transcriptome and H3K4 methylation ChIP-seq data revealed functional divergence among different methylation states in regulating soybean gene expression. Consistent with its established role in maintaining open chromatin and facilitating transcription (Wang et al., 2023; Liu et al., 2024), H3K4me3-marked genes were downregulated upon loss of *GmASH2R*. Conversely, genes bearing H3K4me2 were upregulated in the *ash2r1*/*2* mutants, suggesting that this mark may be associated with transcriptional repression in this context (Liu et al., 2019). This functional divergence among H3K4 methylation states highlights a sophisticated regulatory mechanism through which GmASH2R-containing COMPASS-like complexes fine-tune gene expression across distinct gene sets, enabling soybean to coordinate adaptive responses to developmental and environmental cues.

### GmASH2Rs integrate chromatin modification into the soybean flowering-time regulatory network

We demonstrate that GmASH2Rs directly activate the photoperiodic regulators *GmLHY1a*/*1b*/*2a*/*2b* by promoting H3K4me3 deposition at their promoters. Because *GmLHY*s repress the master flowering repressor *E1*, thereby derepressing the florigen genes *GmFT2a*/*5a* (Dong et al., 2021), our findings define a GmASH2R–GmLHY–E1–FT2a/5a regulatory axis that links chromatin modification with reproductive timing. Notably, GmASH2Rs represent the first identified positive regulators of *GmLHY* expression in legumes, contrasting with the previously reported repressors Tof11 and Tof12 (Lu et al., 2020). Our work demonstrates that COMPASS-like complexes promote flowering in soybean through H3K4me3-dependent transcriptional activation. This epigenetic mechanism, which is fundamentally distinct from the actions of DNA-binding regulators such as J, Tof11, and Tof12 (Lu et al., 2017; Lu et al., 2020), establishes chromatin modification as an essential upstream regulatory layer of the GmLHY–E1–FT2a/5a flowering pathway.

The photoperiod-independent flowering delay and reduced *GmLHY* expression in *ash2r1*/*2* mutants demonstrate that GmASH2R-mediated chromatin activation is required to maintain *GmLHY* transcription. While loss-of-function mutations in *GmLHY*s, particularly combinations containing *lhy1a* (Dong et al., 2021), cause delayed flowering and increased grain yield, the *ash2r1*/*2* double mutant exhibits exacerbated developmental defects, including severe delays in flowering and maturity together with markedly reduced fertility under both LD and SD photoperiods. In Sanya (low-latitude/SD conditions), maturation was delayed by 20 days, but the yield reduction primarily reflected pleiotropic defects in overall plant vigor, reproductive development, and metabolic capacity rather than delayed maturation alone. These pleiotropic defects demonstrate that GmASH2Rs regulate multiple developmental processes beyond the *GmLHY*-mediated flowering pathway, consistent with KEGG enrichment analyses showing the downregulation of photosynthesis, carbon fixation, and porphyrin metabolism genes. The genome-wide impact of *GmASH2R* loss underscores its central role in orchestrating chromatin states across diverse gene networks essential for normal plant growth and reproduction.

### Antagonistic chromatin competition between GmASH2R and GmNF-YC4 proteins establishes dynamic transcriptional control of *GmLHY*s

A notable discovery of this study is the antagonistic regulation of *GmLHY* promoters by GmASH2R and GmNF-YC4 proteins. While GmASH2R2 binds to and activates *GmLHY1a*/*2a* expression via H3K4me3 deposition, GmNF-YC4a competitively binds the same regions to repress transcription. This creates a tunable “Yin–Yang” regulatory module in which the balance between chromatin-modifier-mediated activation and DNA-binding-transcription-factor-mediated repression fine-tunes *GmLHY* expression and flowering time (Figure 7). This regulatory logic fundamentally diverges from that in *Arabidopsis*, where NF-Y complexes interact with CONSTANS (CO) to promote flowering and antagonize Polycomb-mediated H3K27me3 deposition at *FT*, thereby activating *FT* expression (Hou et al., 2014; Liu et al., 2018; Luo et al., 2018). In soybean, GmNF-YC4 functions antagonistically to the COMPASS complex by limiting GmASH2R occupancy, thereby fine-tuning H3K4me3 levels and preventing premature floral transition. Recent studies have revealed that E2/GIGANTEA (GI) and the evening complex form a transcriptional suppression feedback loop that regulates *E1* expression to determine flowering timing and photoperiod sensitivity in soybean (Zhao et al., 2024). Our current study uncovers an epigenetic–transcriptional Yin–Yang regulatory mechanism that operates upstream of *E1* expression through GmLHY (Figure 7). This regulatory inversion may represent a legume-specific rewiring of the flowering network, enabling flexible control of *GmLHY* and dynamic adjustment of flowering time in response to environmental fluctuations.

**Figure 7:**
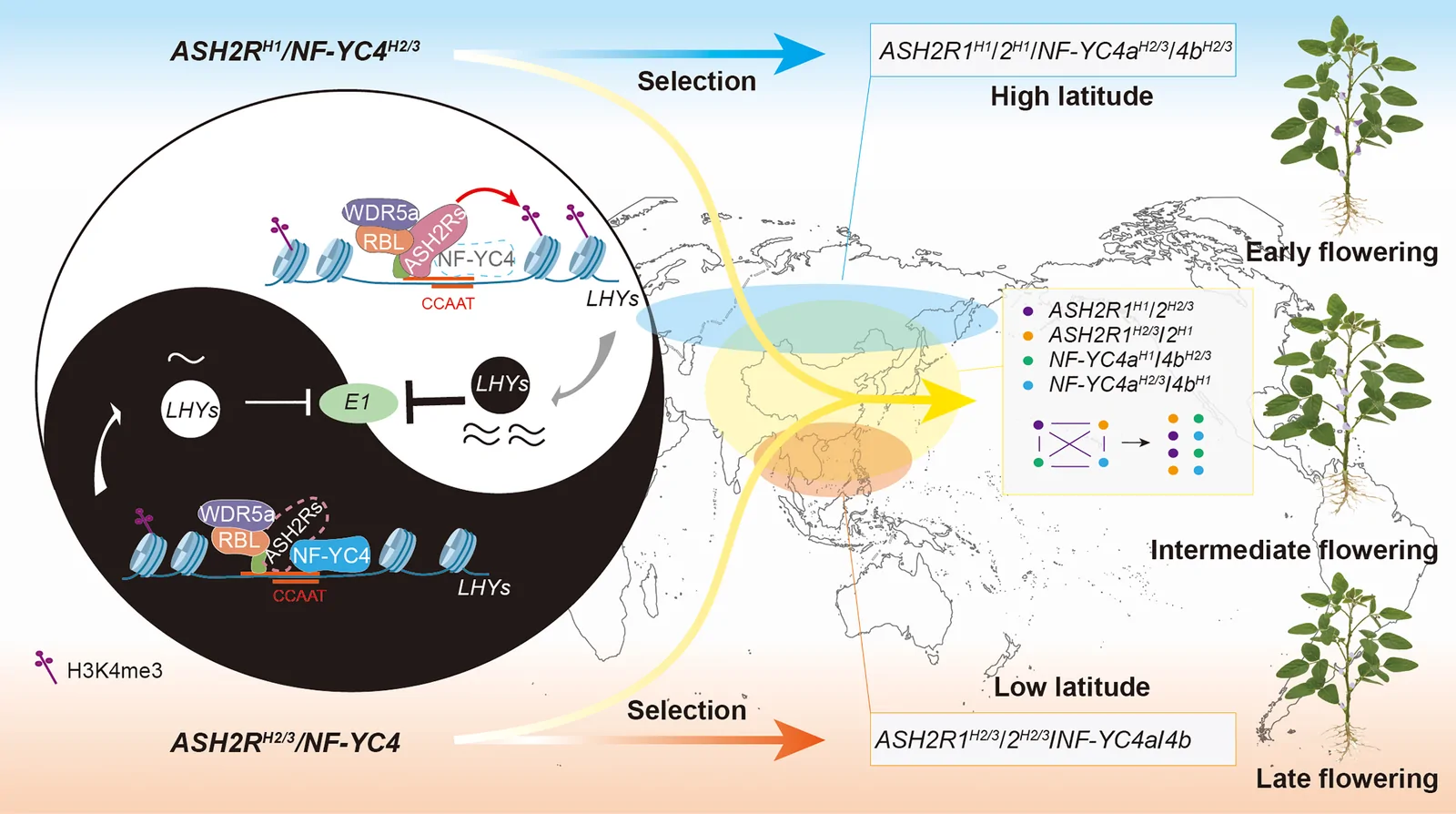
Working model of antagonistic *GmLHY*s regulation by the GmASH2R–COMPASS complex and GmNF-YC4 in soybean flowering adaptation. GmASH2R1/2 forms a COMPASS-like complex with GmRBL and GmWDR5a to activate *GmLHY* expression via H3K4me3 deposition at *GmLHY* promoters, leading to repression of the floral repressor *E1* and accelerated flowering. Conversely, GmNF-YC4a/b compete with this activating complex for access to CCAAT-box motifs within the same regulatory regions, thereby repressing *GmLHY* expression and relieving *E1* repression to delay flowering. These opposing activities establish a “Yin–Yang” homeostatic system that ensures precise control of *GmLHY* levels and flowering time. Throughout soybean domestication and latitudinal expansion, antagonistic selection on these two regulatory components may have generated specific *GmASH2R1*/*2*–*GmNF-YC4a*/*b* allelic combinations that confer differential flowering responses across latitudes. Early-flowering haplotypes *GmASH2R1*^*H1*^/*2*^*H1*^ combined with hypomorphic *GmNF-YC4a*^*H2*/*3*^/4*b*^*H2*/*3*^ were favored in high-latitude environments, whereas *GmASH2R1*^*H2*/*3*^/*2*^*H2*/*3*^ together with *GmNF-YC4a*^*H1*^/4*b*^*H1*^ promotes adaptation to low latitudes. This geographically structured selection may have optimized the epigenetic–transcriptional balance of *GmLHY* expression to adapt flowering time across ecological gradients.

Notably, while GmNF-YC4a was previously reported to repress the florigen genes *GmFT2a*/*5a* (Cai et al., 2024), we now show that GmNF-YC4a and GmNF-YC4b also directly target the upstream regulators *GmLHY*, establishing a multilayered regulatory framework that positions these paralogs as key gatekeepers of soybean flowering. The *nf-yc4a*/*4b* double mutant exhibits significantly earlier flowering than the single mutants under LD but not under SD conditions, contrasting sharply with the photoperiod-independent delay observed in *ash2r1*/*2* mutants. This LD-specific action reveals functional redundancy between the two paralogs and suggests that GmNF-YC4 proteins selectively buffer photoperiodic induction, preventing premature flowering that could compromise vegetative growth and yield. The strict dependence on LDs implies that photoperiod-responsive cofactors, potentially including co-repressors, chromatin remodelers, or factors mediating post-translational modifications, modulate GmNF-YC4 repressive activity in a light-dependent manner. Whether alternative transcription factors recruit GmASH2Rs under SD conditions or at different developmental stages, and how the repressive activity of GmNF-YC4 is photoperiodically controlled, remain important questions for future investigation.

### Antagonistic selection on the GmASH2R1/2–GmNF-YC4a/b module drives soybean latitudinal adaptation

The competitive interaction between GmASH2R and GmNF-YC4 defines a dynamic chromatin-based regulatory circuit in which COMPASS-mediated activation is counterbalanced by NF-YC4-mediated transcriptional repression. This balance enables precise tuning of *GmLHY* expression and, consequently, soybean flowering time. Importantly, population-genetic and evolutionary analyses indicate that this antagonistic regulatory module is not merely a molecular feature but has also been shaped by selection during soybean domestication and improvement, resulting in latitude-associated haplotype combinations that appear to optimize flowering time and promote latitudinal adaptation.

Population genomic analyses reveal sustained and antagonistic selection on *GmASH2R1*/*2* and *GmNF-YC4a*/*b* throughout soybean domestication and improvement. *GmASH2R1* shows sustained high differentiation (*F*_*ST*_ > 0.16) with bottleneck–recovery dynamics, whereas *GmASH2R2* exhibits a stepwise reduction in diversity from wild (π = 0.00187) to cultivar (π = 0.00092) populations (Figures 6A and 6B). Despite these distinct evolutionary trajectories, both *GmASH2R* paralogs display convergent geographic patterns: early-flowering alleles (*GmASH2R1*^*H1*^ and *GmASH2R2*^*H1*^) show higher transcriptional activity and are enriched at high latitudes, whereas late-flowering weak alleles are enriched at low latitudes (Figures 6E, 6F, and 6I). Importantly, although single mutants of *GmASH2R1* or *GmASH2R2* show no flowering-time phenotype due to functional redundancy, natural populations exhibit clear flowering-time differences among these haplotypes (Figures 6G and 6H), likely reflecting the combined effects of complex genetic backgrounds and convergent allele distributions. Strikingly, *GmNF-YC4a*/*b* exhibit an inverse distribution pattern, with late-flowering haplotypes (*GmNF-YC4a*^*H1*^ and *GmNF-YC4b*^*H1*^) prevailing at low latitudes, while early-flowering haplotypes dominate at high latitudes (Supplemental Figures 14A and 14B). This mirror-image distribution between *GmASH2R* and *GmNF-YC4* reflects antagonistic selection that has fine-tuned flowering time by optimizing the opposing activities of these regulators across ecological gradients. Northern varieties employ a dual adaptive strategy that combines GmASH2R1/2-mediated activation of *GmLHY*s with attenuated GmNF-YC4-mediated repression, thereby promoting flowering under LD conditions. In contrast, southern varieties utilize a distinct mechanism that depends more on weak haplotypes of *GmASH2R1*/*2*, with *GmNF-YC4a*/*b* playing a limited repressive role, to prevent premature flowering under SD conditions.

Phenotypic validation suggests that specific allelic combinations optimize latitudinal adaptation: early-flowering haplotypes *GmASH2R1*^*H1*^/*2*^*H1*^, when combined with hypomorphic *GmNF-YC4a*^*H2*/*3*^/4*b*^*H2*/*3*^ alleles, were favored in high-latitude environments. In contrast, the combination of *GmNF-YC4a*^*H1*^/4*b*^*H1*^ with *GmASH2R1*^*H2*/*3*^/*2*^*H2*/*3*^ confers late flowering and occurs predominantly in low latitudes (Figures 6J and 6K). Consistent with this, plants carrying *GmASH2R1*^*H1*^/*2*^*H1*^ together with *GmNF-YC4a*^*H1*^/*b*^*H1*^ flower significantly earlier than those carrying *GmASH2R1*^*H2*/*3*^/*2*^*H2*/*3*^ together with the same *GmNF-YC4a*^*H1*^/*b*^*H1*^ background (Figure 6K). This coordinated selection demonstrates how antagonistically acting regulators generate graded phenotypic variation across ecological ranges without requiring new structural variants. Parallel selection on *GmASH2R1*/*2* and *GmNF-YC4a*/*b* paralogs underscores how gene duplication facilitates trait fine-tuning through allelic diversification rather than dosage effects alone, facilitating soybean expansion from temperate East Asia into tropical and subtropical environments. Because of limitations in the population resources used in this study, we were unable to directly test whether *GmASH2R1*/*2* and *GmNF-YC4a*/*b* exhibit antagonistic expression patterns across diverse germplasm. This question should be addressed in future studies using expanded, geographically diverse populations. In addition, the molecular mechanisms underlying the antagonistic interaction between GmASH2R and GmNF-YC4, as well as its potential conservation across other legumes, remain to be fully elucidated.

In summary, our findings reveal a chromatin–transcriptional “Yin–Yang” module that governs flowering adaptation in soybean (Figure 7). GmASH2R1/2 assemble into a COMPASS-like complex with GmRBL and GmWDR5a to deposit H3K4me3 at *GmLHY* promoters, thereby activating *GmLHY* transcription and promoting flowering. By contrast, GmNF-YC4a/b competitively bind CCAAT-box elements within these promoters to suppress *GmLHY* transcription, thereby establishing an antagonistic regulatory circuit. This balance between chromatin activation and transcriptional repression enables precise, tunable control of *GmLHY* expression and flowering time (Figure 7). Together, these findings suggest that complementary selection acting on these opposing regulatory components during domestication contributed to latitude-specific allelic combinations, driving flowering-time diversification and facilitating the geographic expansion of soybean. This work links epigenetic regulation with adaptive evolution and provides a mechanistic framework for rationally engineering photoperiod-adapted cultivars to accelerate crop improvement.

## Methods

### Plant materials and growth conditions

The soybean (Glycine *max* (L.) Merr.) cultivar Williams 82 was used in this study. Field-grown plants were cultivated under natural LD conditions in Weifang (36°59′ N, 119°42′ E; June–October) or under natural SD conditions in Sanya (18°38′ N, 109°15′ E; November–March). For controlled-environment studies, plants were grown in growth chambers under LD (16 h light/8 h darkness) or SD (12 h light/12 h darkness) conditions at an irradiance of approximately 400 μmol m^−2^ s^−1^, 25°C, and 60% relative humidity. For RNA-seq and ChIP-qPCR experiments, leaves were collected from 3-week-old plants at ZT4 under LD conditions. For phenotypic analysis, the R1 stage was recorded as the number of days from emergence to the first open flower at any main-stem node, and the R8 stage was recorded as the number of days from emergence to complete pod browning (Dong et al., 2022).

### Generation of mutants and overexpression transgenic lines

For CRISPR-Cas9-mediated gene editing, guide RNA sequences targeting *GmASH2R1*, *GmASH2R2*, *GmNF-YC4a*, and *GmNF-YC4b* were designed using the CRISPR-P 2.0 web tool (http://crispr.hzau.edu.cn/cgibin/CRISPR2/CRISPR). Oligonucleotides corresponding to the guide RNAs targeting *GmASH2R1* and *GmASH2R2* were synthesized and cloned into BsaI-digested *pGES201* (Bai et al., 2020), while those targeting *GmNF-YC4a* and *GmNF-YC4b* were amplified and cloned into *p007,* a modified *pGES401* vector (Bai et al., 2020). *p007* was generated by inserting a 35S-driven spectinomycin-resistance cassette into the StuI site of pGES401.

To generate overexpression constructs, the full-length coding sequences of *GmASH2R1*, *GmASH2R2*, and *GmNF-YC4a*, excluding stop codons, were amplified, cloned into *pENTR3-N3×FLAG*, and subsequently transferred into *pB2GW7* via Gateway recombination (Curtis and Grossniklaus, 2003). All constructs were confirmed by Sanger sequencing and introduced into *Agrobacterium tumefaciens* strain EHA105. Soybean transformation was performed using cotyledon explants as previously described (Olhoft et al., 2006). Transgenic plants were confirmed by western blot analysis.

### Yeast one-hybrid assay

For Y1H assays, the full-length coding sequence of *GmNF-YC4a* was cloned into *pJG5*, which contains the GAL4 activation domain, and promoter fragments of *GmLHY1a* and *GmLHY2a* were cloned into *pLacZi* (Li et al., 2022). The recombinant plasmids were co-transformed into *Saccharomyces cerevisiae* strain EGY48, and transformants were grown on synthetic defined medium lacking tryptophan and uracil (SD/−Trp/−Ura) for 48 h. Transformants were then spotted onto selective medium lacking tryptophan and uracil and supplemented with galactose, raffinose, and X-Gal (SC/−Trp/−Ura/Gal/Raf/X-Gal) to assess reporter gene expression (Coolaber, YK3010-50P). Empty *pJG5* and *pLacZi* vectors were used as negative controls.

### Yeast two-hybrid assay

For Y2H assays, full-length coding sequences were amplified from Williams 82 cDNA and cloned into *pGADT7* (prey) and *pGBKT7* (bait) vectors (Clontech). Plasmid pairs were co-transformed into *S. cerevisiae* strain AH109 (Clontech) and selected on SD medium lacking leucine and tryptophan (SD/−Leu/−Trp). Protein–protein interactions were assessed by growth on highly selective solid dropout medium lacking leucine, tryptophan, histidine, and adenine (SD/−Leu/−Trp/−His/−Ade).

### Bimolecular fluorescence complementation assays

For BiFC assays, the full-length coding sequences of *GmASH2R1/2*, *GmRBL1/2/3*, and *GmWDR5a1* were cloned into *pCAMBIA1300-nYFP* or *pCAMBIA1300-cYFP* to generate fusion constructs containing the N-terminal fragment of eYFP (amino acids 1–173) or the C-terminal fragment of eYFP (amino acids 155–239), respectively (Kodama and Hu, 2010). Paired plasmids were introduced into *N. benthamiana* epidermal cells by *Agrobacterium tumefaciens* strain GV3101-mediated transient expression. At approximately 48 h post-infiltration, eYFP fluorescence signals were visualized using a confocal laser scanning microscope (Nikon, A1HD25-SIM S).

### Luciferase complementation imaging assay

The full-length coding sequence of *GmASH2R2 was cloned into pCAMBIA1300-cLUC, and the f*ull-length coding sequences of *GmNF-YC4a*/*b* were cloned into *pCAMBIA1300-nLUC* (Chen et al., 2008). Paired constructs were co-infiltrated into *N. benthamiana* leaves using *A. tumefaciens* strain GV3101. Luciferase signals were detected at 48 h post-infiltration using a ChemiDoc MP imaging system (Bio-Rad).

### Subcellular localization assays

The full-length coding sequences of *GmASH2R1*/*2*, *GmRBL1*/*2*/*3*, and *GmWDR5a1*/*a2* were fused to GFP in the *pSuper-GFP* vector (Wang et al., 2025a) and transiently expressed in *N. benthamiana* leaves using *A. tumefaciens* strain GV3101. GFP signals were visualized at 48–72 h post-infiltration by confocal microscopy (Nikon, A1HD25-SIM S).

### Co-immunoprecipitation assays

Co-IP experiments were performed as described previously, with minor modifications (Wang et al., 2014). For assays performed in soybean hairy roots, the full-length coding sequences of *GmASH2R1* and *GmWDR5a1* were fused to *3×FLAG*, and the full-length coding sequence of *GmRBL1* was fused to *3×HA.* These constructs were cloned into *pCAMBIA1300* and introduced into soybean cotyledons using *Agrobacterium rhizogenes* strain K599 to generate transgenic hairy roots (Fan et al., 2020). For transient-expression assays in *N. benthamiana*, *GmASH2R2* was fused to *3×FLAG*, and *GmNF-YC4a* and *GmNF-YC4b* were fused to *3×HA*; the resulting constructs were cloned into *pB2GW7* (Curtis and Grossniklaus, 2003). Paired constructs were co-infiltrated into *N. benthamiana* leaves using *A. tumefaciens* strain GV3101. Transgenic hairy roots (0.5 g) or infiltrated leaves (1.0 g) were ground in liquid nitrogen and extracted in IP buffer (50 mM Tris–HCl [pH 7.4], 100 mM NaCl, 1 mM EDTA, 0.1% [v/v] NP-40, 1 mM PMSF, and 1× protease inhibitor cocktail [Roche]). Protein extracts (1.0 ml) were incubated with 20 μl of Anti-DYKDDDDK Magnetic Agarose beads (Invitrogen, A36798) at 4°C. Immunoprecipitated proteins were detected by western blotting using anti-FLAG (Sigma-Aldrich, A8592, 1:2,000) and anti-HA (Roche, 12013819001, 1:2,000) antibodies.

### Gene expression analysis

Total RNA was extracted from the fully expanded trifoliate leaves of seedlings grown for 3 weeks under LD conditions or 2 weeks under SD conditions using the RNeasy Plus Mini Kit (QIAGEN, 74134). RNA was reverse-transcribed using HiScript III RT SuperMix for qPCR (Vazyme, R323-01). RT-qPCR was performed using SYBR Green Master Mix (Invitrogen, A25742) on a QuantStudio 5 system (Applied Biosystems). The constitutively expressed *Tubulin* gene (*Glyma.05G157300*) served as an internal control for normalization, and relative transcript levels were calculated using the 2^−ΔCt^ method. Three biologically independent samples were analyzed for each experiment. Primers are listed in Supplemental Table 1.

### H3K4 methylation level analysis

Histone extraction and western blot analysis were performed as previously described (Gu et al., 2009). Briefly, total histones were extracted from the leaves of 3-week-old seedlings grown under LD conditions, separated by SDS-PAGE, and transferred to 0.45-μm polyvinylidene fluoride membranes (Merck Millipore). Membranes were probed with antibodies against H3K4me1 (Millipore, 07-436, 1:2,000), H3K4me2 (Millipore, 07-030, 1:2,000), H3K4me3 (Millipore, 07-473, 1:2,000), and H3 (Abcam, ab1791, 1:10,000). Two biological replicates were analyzed for each mutant.

### Chromatin immunoprecipitation

ChIP experiments were performed as previously described, with modifications (Saleh et al., 2008; Li et al., 2021). Briefly, fully expanded trifoliate leaves were harvested at ZT4 under LD conditions from 3-week-old WT Williams 82 (Wm82), mutant (*ash2r 1-1*/*2-1* and *nf-yc4a*/*4b-1*), or transgenic plants expressing *35Spro:GmASH2R2-FLAG* or *35Spro:GmNF-YC4a-FLAG*. For transient expression assays, *35Spro:GmNF-YC4a-FLAG* or *35Spro:GmASH2R2-FLAG* was introduced into *A. tumefaciens* GV3101 and infiltrated into the leaves of 3-week-old WT or mutant plants (*ash2r1*/*2* and *nf-yc4a*/*b*); empty vector (*35Spro:FLAG*) served as the control. Leaves were harvested 48 h post-infiltration at ZT4. Samples were ground in liquid nitrogen, suspended in ChIP buffer, and cross-linked with 1% formaldehyde for 15 min at room temperature. Reactions were quenched with 2 M glycine, followed by nuclei isolation. Chromatin was sonicated into 200- to 750-bp fragments using a Bioruptor (Diagenode). Immunoprecipitation was carried out with anti-FLAG (Sigma, F1804, 1:575) or anti-H3K4me3 (Millipore, 07-030, 1:575) antibodies using protein A/G magnetic beads (Merck Millipore). Enriched DNA was quantified by qPCR using SYBR Green Master Mix (Invitrogen, A25742) on a QuantStudio 5 system (Applied Biosystems). Three biological replicates were analyzed for each experiment. For target-protein ChIP, relative enrichment at each examined region was first normalized to the endogenous control *Tubulin* (*Glyma.05G157300*) and then to the corresponding WT control. For histone-modification ChIP, enrichment at each examined region was normalized directly to input. Primers are listed in Supplemental Table 1.

### *Agrobacterium*-mediated transient transformation in soybean leaves

Transient expression assays were performed as previously described, with modifications (Li et al., 2019; Guo et al., 2025). *A. tumefaciens* strain GV3101 carrying target constructs or the empty-vector control was cultured in LB medium at 28°C and 200 rpm for 12–16 h. Cells were collected and resuspended in infiltration buffer (10 mM MES, 10 mM MgCl_2_, and 100 μM acetosyringone [pH 5.6]) to an OD_600_ of 1.0. Leaf discs (8-mm diameter) from 3-week-old WT or mutant plants were submerged in bacterial suspension and vacuum infiltrated for 10–15 min. Infiltrated discs were incubated on moistened filter paper at 28°C in darkness for 24 h, followed by an additional 24 h under LD conditions in a growth chamber. Samples were then harvested for subsequent ChIP analysis.

### Electrophoretic mobility shift assay

The full-length coding sequence of *GmNF-YC4a* was cloned into *pET28a-His* and transformed into *Escherichia coli* BL21 (DE3). Fusion proteins were induced with 10 mM Isopropyl-beta-D-thiogalactopyranoside (IPTG) at 16°C for 16–20 h and purified using a His-tag affinity purification kit (Cytiva, 10310519). Biotin-labeled and unlabeled probes containing putative binding sites from the *GmLHY1a* and *GmLHY2a* promoters were synthesized (RuiBiotech) and annealed. EMSA was performed using the Chemiluminescent EMSA Kit (Beyotime, GS009), and signals were detected with a ChemiDoc MP System (Bio-Rad). Three biological replicates were analyzed.

### Luciferase-based transient transcriptional activity assay

The full-length coding sequences of *GmNF-YC4a*/*b* were cloned into the *p1300-35S:GFP* vector to generate *35S:GmNF-YC4a*/*b-GFP* effector constructs. For reporter constructs, the 1.6- and 1.8-kb promoter regions of *GmLHY1a* and *GmLHY2a*, respectively, were amplified and cloned into the *pFA2300-LUC* vector (Li et al., 2022). The effector and reporter plasmids were introduced into *A. tumefaciens* GV3101 and co-infiltrated into *N. benthamiana* leaves. At 48 h post-infiltration, plants were transferred to darkness at 22°C before luciferase imaging. Luciferase activity was quantified using a ChemiDoc MP Imaging System (Bio-Rad). At least three independent biological replicates were performed for each experiment.

### Dual-luciferase reporter assay

Dual-luciferase reporter assays were performed as described previously (Hellens et al., 2005). For reporter construct generation, promoter fragments representing distinct *GmASH2R1* haplotypes (−1997 to −1 bp relative to the start codon) and *GmASH2R2* haplotypes (−1977 to −1 bp) were amplified and cloned into the *pGreenII 0800-LUC* vector. *A. tumefaciens* strain GV3101 carrying the corresponding reporter constructs was infiltrated into *N. benthamiana* leaves. LUC and *Renilla* luciferase activities were measured 48–72 h post-infiltration using the Dual-Luciferase Reporter Assay System (Promega, E1910) according to the manufacturer’s instructions. Relative promoter activity was calculated as the LUC/*Renilla luciferase* luminescence ratio for each treatment.

### Genetic diversity and haplotype analysis

Whole-genome resequencing data for all accessions were obtained from the Soybean Genomic Variation Database (https://yanglab.hzau.edu.cn/SoyGVD/). Single-nucleotide polymorphisms with a missing rate greater than 10% or a minor allele frequency less than 5% were removed from VCF files using BCFtools (v1.20). Single-nucleotide polymorphisms within gene bodies and 2.5-kb upstream promoter regions were extracted for haplotype classification. Haplotype structures were analyzed and visualized using the R package geneHapR (v1.2.4). Only haplotypes with a frequency > 5 or those closely related to major haplotypes were included in downstream analyses. Population differentiation statistics, including *F*_*ST*_ and nucleotide diversity (*π*), were calculated using VCFtools (v0.1.16) with 100-kb sliding windows and 10-kb step sizes across wild, landrace, and improved soybean populations. Regional classification of soybean accessions was based on a previous description (Li et al., 2010). The map of China was obtained from the Ministry of Natural Resources of China (https://www.bzdt.ch.mnr.gov.cn).

### RNA sequencing data analysis

Total RNA was extracted from samples and subjected to RNA-seq. Sequencing reads were aligned to the soybean reference genome Wm82.a6.v1 using HISAT2 (v2.2.1), and gene-level read counts were quantified with featureCounts (v1.6.3). DEGs were identified using DESeq2 (v1.48.1) with thresholds of |log_2_(FC)| ≥ 1 and a false discovery rate (FDR) < 0.05. Gene Ontology (GO) and KEGG enrichment analyses were performed using clusterProfiler (v4.16.0).

### ChIP-seq data analysis

ChIP-seq data were retrieved from the Genome Sequence Archive (CRA019775, https://ngdc.cncb.ac.cn/) (Liu et al., 2025). Raw reads were quality-filtered using fastp (v0.23.2) and aligned to the soybean Williams 82 reference genome assembly Wm82.a6.v1 using Bowtie2 (v2.5.1). PCR duplicates were removed with Picard (v2.23.3). Reads carrying the “XS:” tag in the SAM file, which indicates multiple alignments, were excluded from downstream analyses. Peaks were called using MACS3 (v3.0.3) with the following parameters: -f BAMPE -min-length 100 -g 1e9 -B -q 0.05. Peaks were annotated using the R package ChIPseeker.

### Data availability

All data supporting the findings of this study are included within the article and its supplemental information files or are available from the corresponding author upon reasonable request. The bulk RNA-seq data generated in this study have been deposited in the Genome Sequence Archive (GSA) at the National Genomics Data Center under accession no. CRA070096. The ChIP-seq data analyzed in this study were retrieved from the GSA under accession no. CRA019775.

## Funding

This work was supported by the 10.13039/501100012166National Key R&D Program of China (2024YFF1000300 to X. Luo), the 10.13039/501100001809National Natural Science Foundation of China (32570416 to X. Luo), the National High-Level Talents Special Support Plan (to X. Luo), the Natural Science Foundation of Shandong Province (ZR2025QC248 to C. Huang and ZR2022QC262 to X. Liu, project SYS202206), the Taishan Scholars Program (to X. Luo), the Yuandu Scholars Program (to X. Luo), and the Excellent Research Group Project of the 10.13039/501100001809National Natural Science Foundation of China (grant no. 32488102 to X.W.D.).

## Acknowledgments

We thank Professor Yuefeng Guan from Guangzhou University for kindly sharing the original CRISPR-Cas9 vectors for soybean gene editing. We are also grateful to the Biotechnology Platform and the Bioinformatics Platform of the 10.13039/501100007937Peking University Institute of Advanced Agricultural Sciences for their assistance with soybean genetic transformation and computational support, respectively. No conflict of interest is declared.

## Author contributions

X. Luo conceived and designed the research. N.Z. and C.H. performed most experiments, and C.S. analyzed the bioinformaticdata. X.M., W.R., X. Liu, and Z.Z. assisted with the generation and transformation of genetic material, and L.F., S.Z., and X.W.D. provided technical support and suggestions. X. Luo, N.Z., C.H., and C.S. interpreted the results. X. Luo wrote the manuscript with input from N.Z. and C.S.

## Supplemental information

Document S1. Supplemental Figures 1–14 and Supplemental Table 1

Document S2. Article plus supplemental information
