# Supplementary material for: Antagonistic chromatin regulation of LHY homologs by GmASH2R and GmNF-YC4 fine-tunes soybean flowering time for latitudinal adaptation
Source: Plant Commun. 2026 May 20;7(10):101917. doi: 10.1016/j.xplc.2026.101917 (PMC13681401; doi:10.1016/j.xplc.2026.101917)
Supplement: Supplementary file 1 [file mmc1.pdf]

Plant Communications, Volume *7*

Supplemental information

Antagonistic chromatin regulation of *LHY* homologs by GmASH2R and GmNF-YC4 fine-tunes soybean flowering time for latitudinal adaptation

Na Zheng, Can Huang, Chengyang Song, Xiaofei Ma, Wenwen Ren, Xiulin Liu, Zhaohan Zhang, Lingyang Feng, Shangwei Zhong, Xing Wang Deng, and Xiao Luo

Supplemental Information

Supplemental Figure 1. Protein sequence comparisons of ASH2R in Arabidopsis and Soybean.

Supplemental Figure 2. Characterization of the *GmASH2R1* and *GmASH2R2* in soybean.

Supplemental Figure 3. Gene structures and CRISPR/Cas9-induced mutations of *GmASH2R1* and *GmASH2R2* in soybean.

Supplemental Figure 4. Flowering phenotypes of *GmASH2R1* and *GmASH2R2* overexpression lines under short-days (SDs) and long-days (LDs).

Supplemental Figure 5. Evolutionary analysis and subcellular localization of GmRBL and GmWDR5a homologs.

Supplemental Figure 6. GmASH2R and GmWDR5a proteins interact with GmRBL homologs in yeast and plant cells.

Supplemental Figure 7. Yeast two hybrid assay of the interaction between GmASH2Rs and GmWDR5as homologs.

Supplemental Figure 8. Transcriptomic profiling of *ash2r1/2* double mutants.

Supplemental Figure 9. Expression analysis of flowering pathway genes in *ash2r1/2* mutants under short-days (SDs).

Supplemental Figure 10. Generation and verification of *GmNF-YC4a* and *GmNF-YC4b* mutant lines using CRISPR/Cas9.

Supplemental Figure 11. GmNF-YC4a and GmASH2R2 antagonistically regulate *GmLHY* promoter activity.

Supplemental Figure 12. Western blot analysis of GmNF-YC4a and GmASH2R2 in soybean overexpression lines and transient transformation assays.

Supplemental Figure 13. Haplotype analysis and evolutionary origins of *GmASH2R1/2* and *GmNF-YC4a/b* in soybean populations.

Supplemental Figure 14. Geographic distribution and flowering time associations of *GmNF-YC4a/b* haplotypes link to their antagonistic *GmASH2R1/2* alleles in soybean.

Supplementary Table 1: Primers used in this study

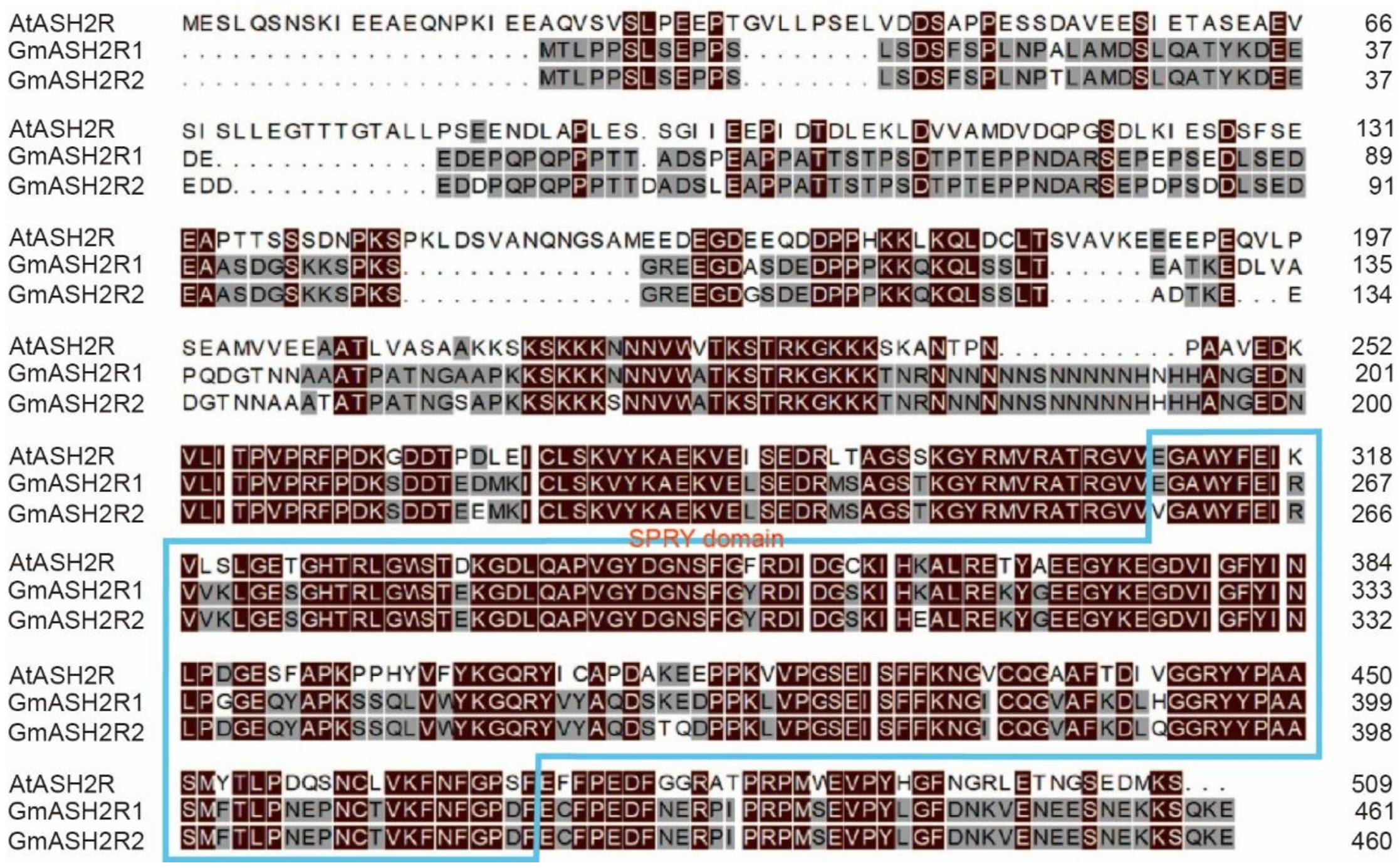

Supplemental Figure 1. Protein sequence comparisons of ASH2R in Arabidopsis and Soybean. Identical residues are shaded in black, while similar residues are shaded in gray. Blue frame indicates the SPla/RYanodine receptor (SPRY) domain.

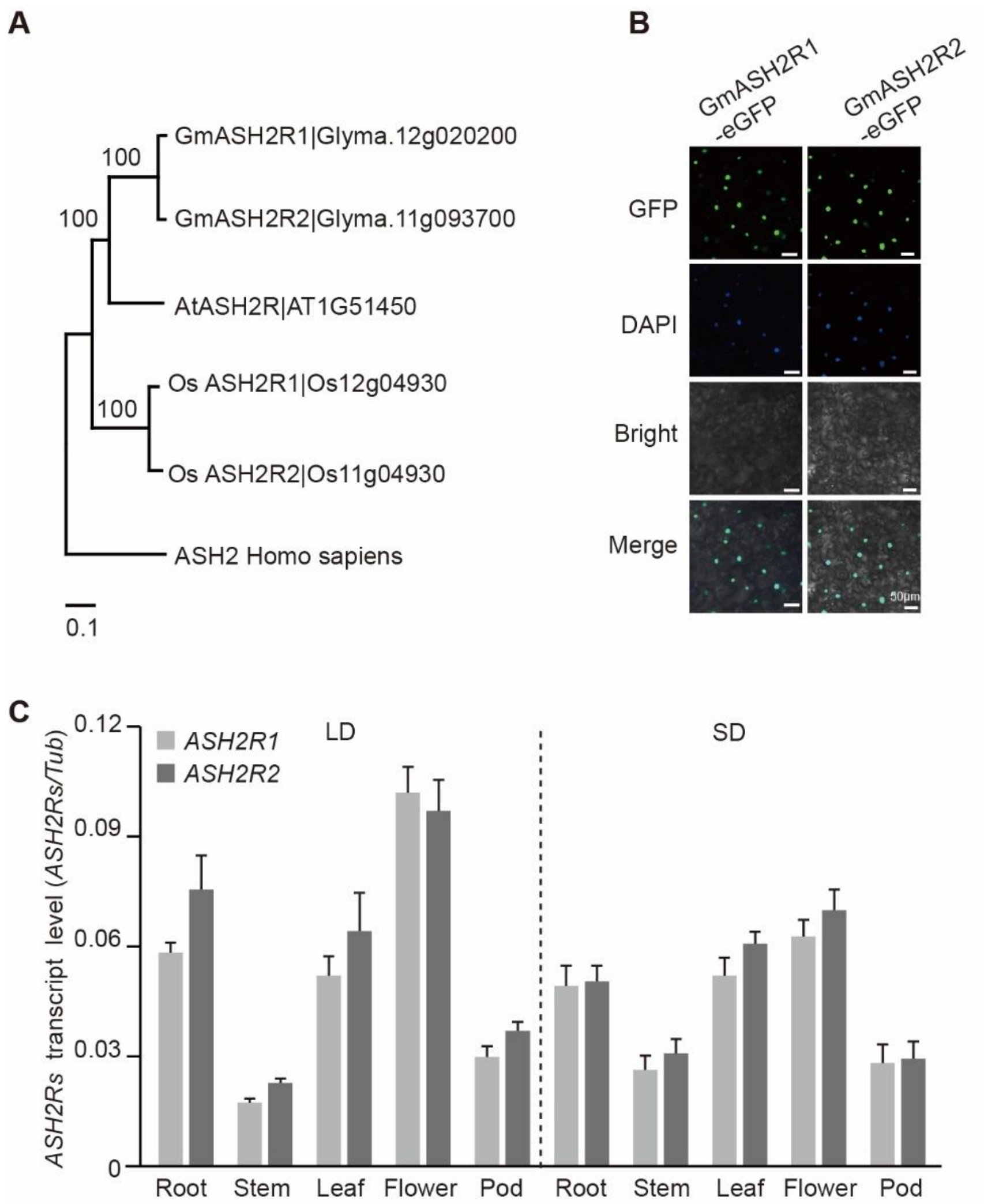

Supplemental Figure 2. Characterization of the *GmASH2R1* and *GmASH2R2* in soybean. (A) Phylogenetic analysis of ASH2R homologs. The neighbor-joining tree was constructed in MEGA-X with 1000 bootstrap replicates. Numbers at nodes indicate bootstrap support values. Scale bar represents 0.1 amino acid substitutions per site. **(B)** Nuclear localization of GmASH2R1 and GmASH2R2 in *N.benthamiana* leaves epidermal cells. GFP-tagged GmASH2R1 and GmASH2R2 (green) localize exclusively to nuclei, counterstained with 4′,6-diamidino-2-phenylindole (DAPI) (blue). Scale bars: 50 µm. **(C)** Expression profiles of *GmASH2R1* and *GmASH2R2* across soybean tissues under different photoperiods. Transcript levels of *GmASH2R1* and *GmASH2R2* in roots, stems, leaves, flowers, and pods under long-days (LDs) and short-days (SDs) were quantified by RT–qPCR. All expression values were normalized to the endogenous reference gene *Tubulin.* Data represent means ± standard deviation (s.d.) of six biological replicates.

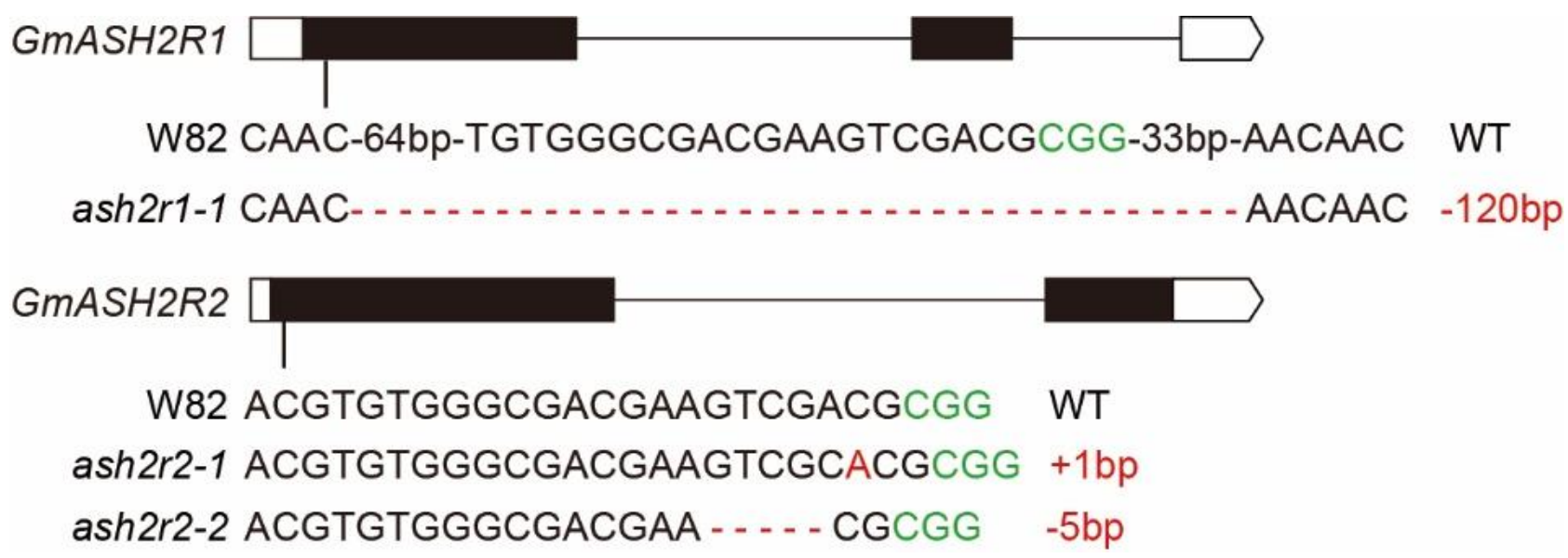

Supplemental Figure 3. Gene structures and CRISPR/Cas9-induced mutations of *GmASH2R1* and *GmASH2R2* in soybean. Schematic diagrams illustrate the exon–intron structures of *GmASH2R1* and *GmASH2R2* (white boxes: UTRs; dark boxes: exons; black lines: introns). Target sequences of sgRNAs are shown below, with protospacer-adjacent motifs (PAM) highlighted in green. Sequence alignments compare the WT (W82) and mutant alleles; deleted and inserted nucleotides are marked by red dashes and letters, respectively. The sizes of indels are indicated on the right.

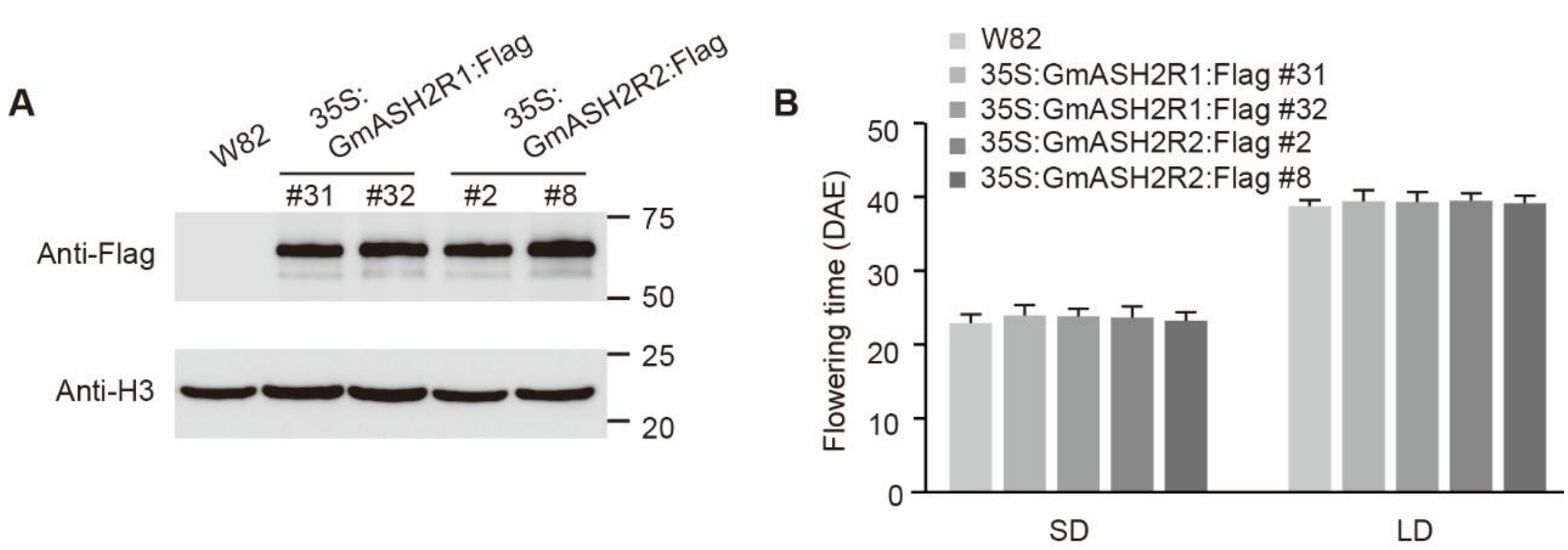

Supplemental Figure 4. Flowering phenotypes of *GmASH2R1* and *GmASH2R2* overexpression lines under short-days (SDs) and long-days (LDs). (A) Immunoblot analysis of GmASH2R1-3×FLAG and GmASH2R2-3×FLAG fusion proteins in transgenic soybean lines. Protein extracts from leaves of overexpression lines grown under LDs were probed with anti-FLAG antibody. Specific bands corresponding to GmASH2R1-3×FLAG and GmASH2R2-3×FLAG. Histone H3 served as a loading control. **(B)** Flowering time evaluation of overexpression lines under SDs and LDs. Days to flowering were recorded for GmASH2R1-3×FLAG and GmASH2R2-3×FLAG overexpression lines under SDs and LDs. Data represent mean ± s.d. (n ≥ 10 plants per line).

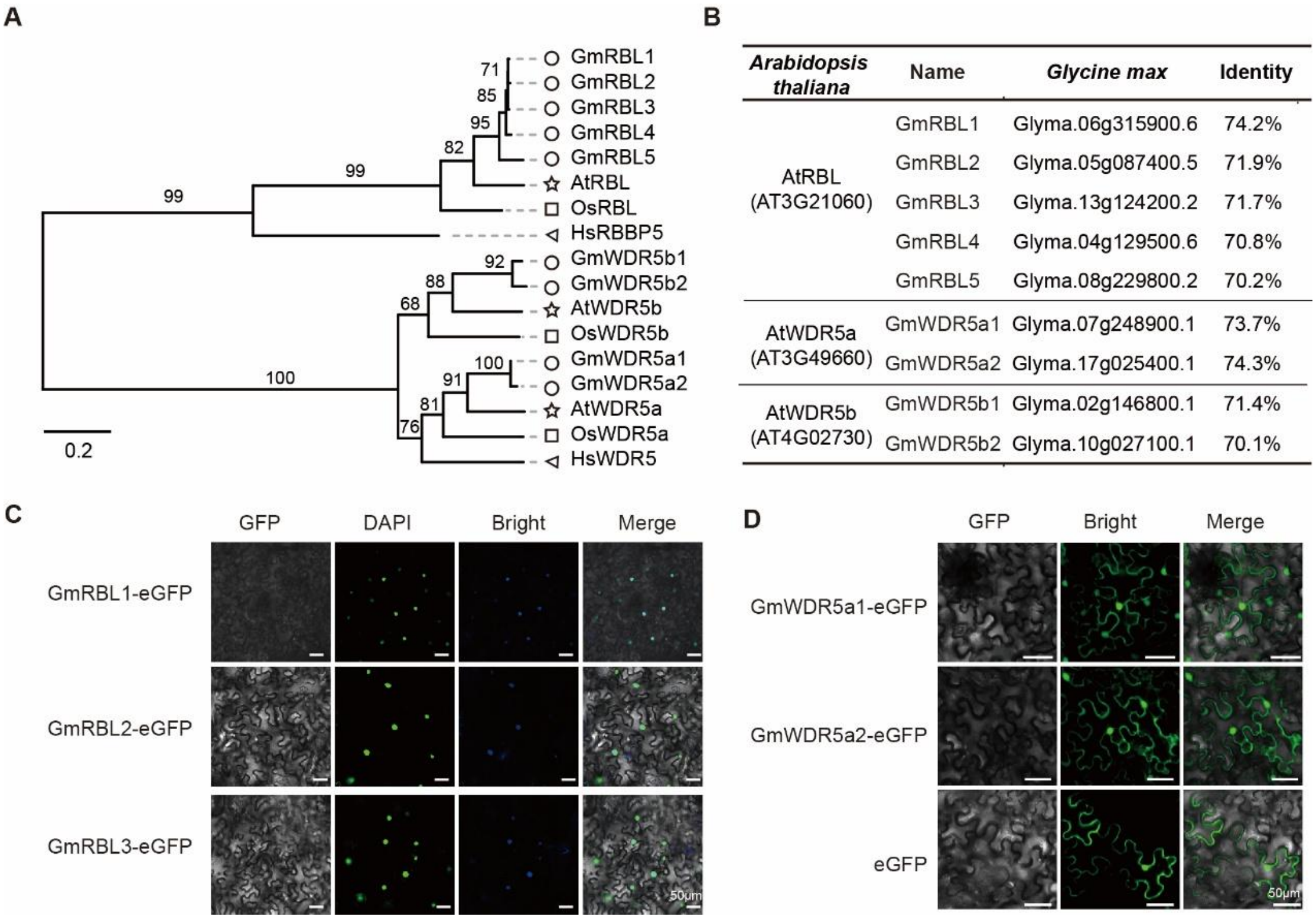

Arabidopsis thaliana	**Name**	***Glycine max***	**Identity**
AtRBL (AT3G21060)	GmRBL1	Glyma.06g315900.6	74.2%
	GmRBL2	Glyma.05g087400.5	71.9%
	GmRBL3	Glyma.13g124200.2	71.7%
	GmRBL4	Glyma.04g129500.6	70.8%
	GmRBL5	Glyma.08g229800.2	70.2%
AtWDR5a (AT3G49660)	GmWDR5a1	Glyma.07g248900.1	73.7%
	GmWDR5a2	Glyma.17g025400.1	74.3%
AtWDR5b (AT4G02730)	GmWDR5b1	Glyma.02g146800.1	71.4%
	GmWDR5b2	Glyma.10g027100.1	70.1%

Supplemental Figure 5. Evolutionary analysis and subcellular localization of GmRBL and GmWDR5a homologs. (A) Phylogenetic analysis of RBL and WDR5a proteins. Evolutionary relationships were inferred using the neighbor-joining method in MEGA-X with 1000 bootstrap replicates. Numbers at nodes indicate bootstrap support values. The scale bar represents 0.2 amino acid substitutions per site. **(B)** Sequence identity among RBL and WDR5a homologs in soybean and Arabidopsis. **(C-D)** Subcellular localization of GmRBL **(C)** and GmWDR5a **(D)** proteins in *N. benthamiana* leaf epidermal cells. GFP-tagged proteins (green) and DAPI-stained nuclei (blue) are shown. Empty GFP vector was used as a localization control. Scale bars: 50 μm.

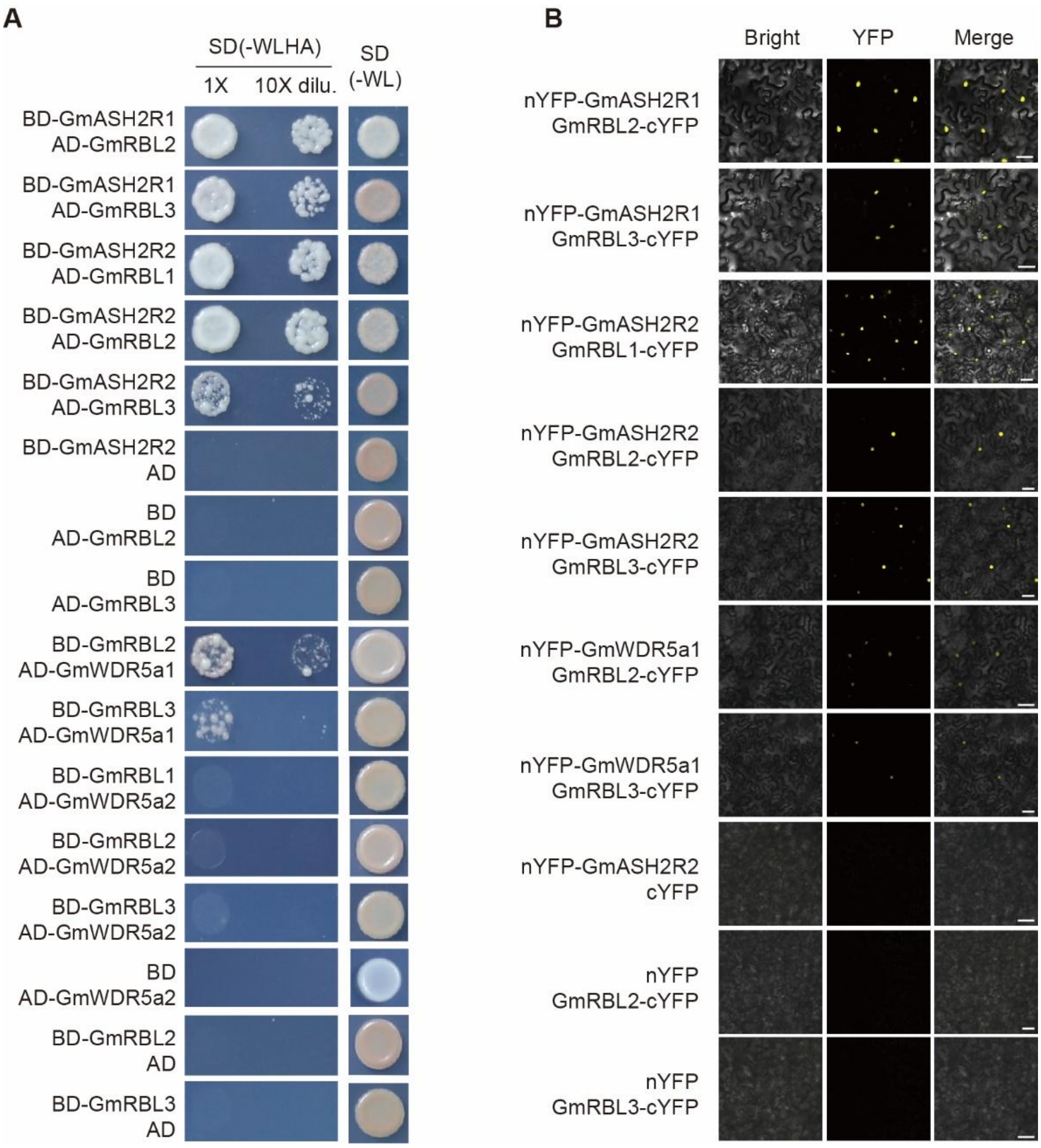

Supplemental Figure 6. GmASH2R and GmWDR5a proteins interact with GmRBL homologs in yeast and plant cells. (A) Yeast two-hybrid assays showing interactions of GmRBL2 and GmRBL3 with GmASH2R1, GmASH2R2 and GmWDR5a1. Yeast cells (undiluted and 10× diluted) were spotted onto stringent selective medium lacking tryptophan, leucine, histidine, and adenine (-WLHA). Control plates lacking only tryptophan and leucine (-WL) confirmed normal growth. **(B)** BiFC validation of GmRBL2 and GmRBL3 interactions with GmASH2R1, GmASH2R2, and GmWDR5a1 in *N. benthamiana* nuclei. Reconstituted YFP fluorescence (yellow) indicates protein-protein interactions. Scale bars: 50 µm.

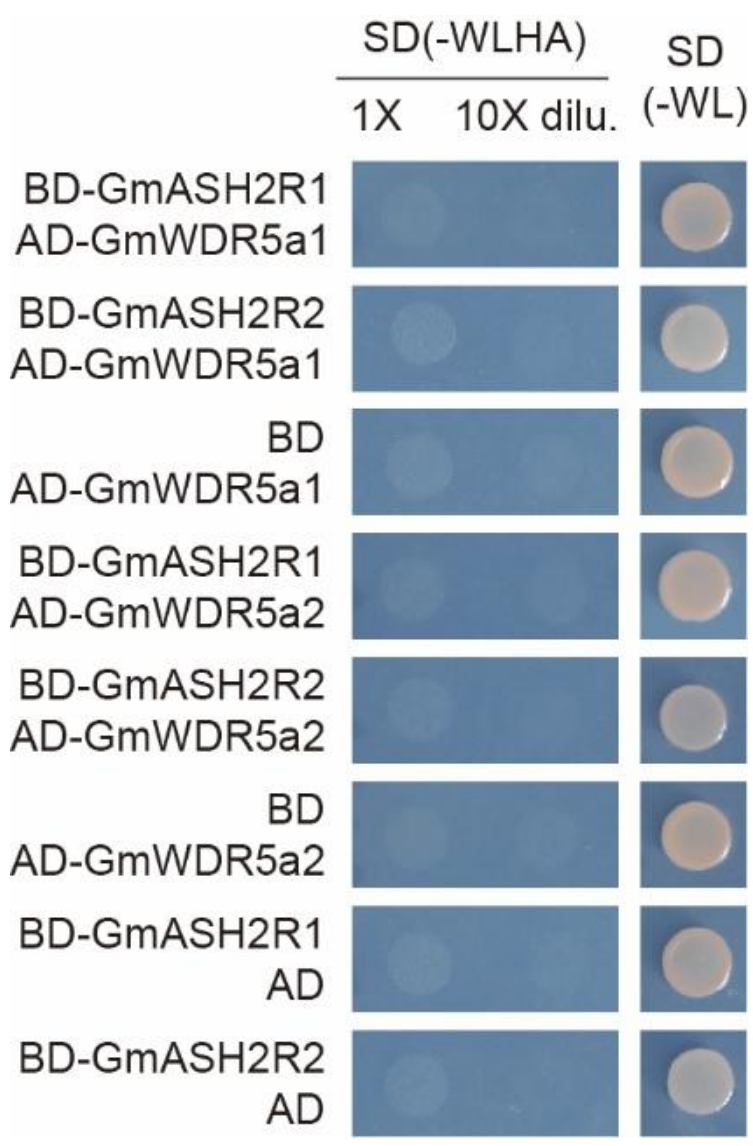

Supplemental Figure 7. Yeast two-hybrid assay of the interaction between GmASH2Rs and GmWDR5as homologs. Yeast two-hybrid assays showing interactions for GmASH2R1/GmASH2R2 and GmWDR5a1/GmWDR5a2.Yeast cells (undiluted and 10× diluted) were spotted onto stringent selective medium lacking tryptophan, leucine, histidine, and adenine (-WLHA). Control plates lacking only tryptophan and leucine (-WL) confirmed normal growth.

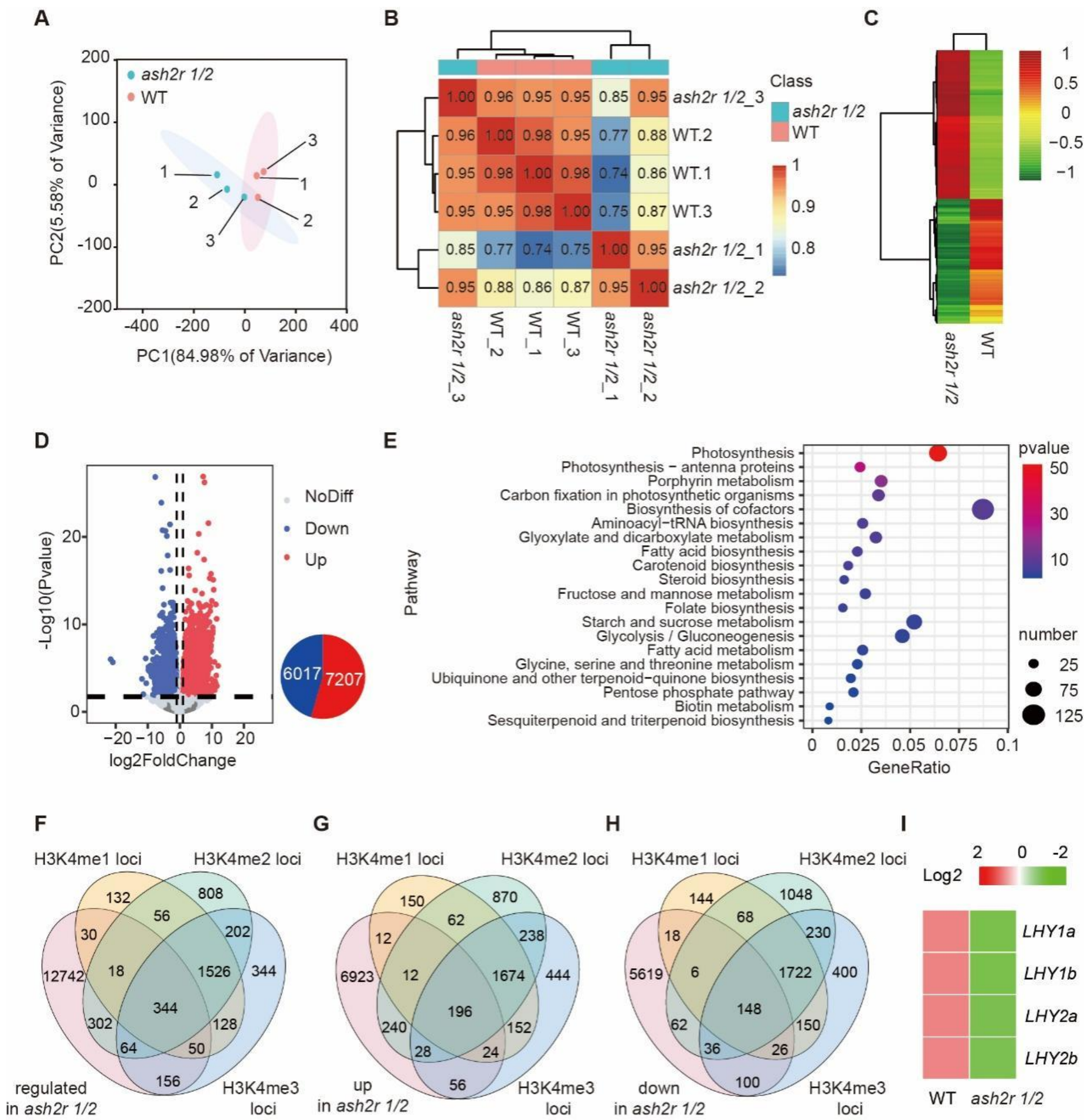

Supplemental Figure 8. Transcriptomic profiling of *ash2r1/2* double mutants. (A) Principal Component Analysis (PCA) of RNA-seq data from *ash2r 1-1/2-1* double mutants and WT (W82). Axes indicate the first (PC1) and second (PC2) principal components. **(B)** Correlation heatmap of transcriptome samples. Sample *ash2r1/2*_3 showed higher correlation with the wild-type group (0.95–0.96) than with its replicate group *ash2r1/2*_1 and _2 (0.85 and 0.95, respectively) and was therefore excluded from subsequent analyses. **(C)** Heatmap of normalized gene expression levels across genotypes. Values represent mean expression of biological replicates. **(D)** Volcano plot of differentially expressed genes (DEGs). Genes with $|\log_2(\text{fold change})| \geq 1$ and FDR < 0.05 in *ash2r1/2* double mutants compared to wild-type W82 are highlighted in red (upregulated) and blue (downregulated). The x-axis denotes $\log_2$(fold change),

and the y-axis indicates the mean gene expression level. The accompanying pie chart summarizes DEG numbers. **(E)** Bubble plot of KEGG pathway enrichment analysis for downregulated genes in *ash2r1/2* double mutants versus wild-type W82. Bubble size corresponds to the number of genes, and color indicates $-\log_{10}$(*p-value*). **(F-H)** Overlap between DEGs and H3K4 methylation marks. Venn diagrams show intersections of all DEGs (F), upregulated genes (G), and downregulated genes (H) with genomic regions marked by H3K4me1, H3K4me2, and H3K4me3. **(I)** Expression heatmap displaying $\log_2$(FPKM) values for *GmLHY*s.

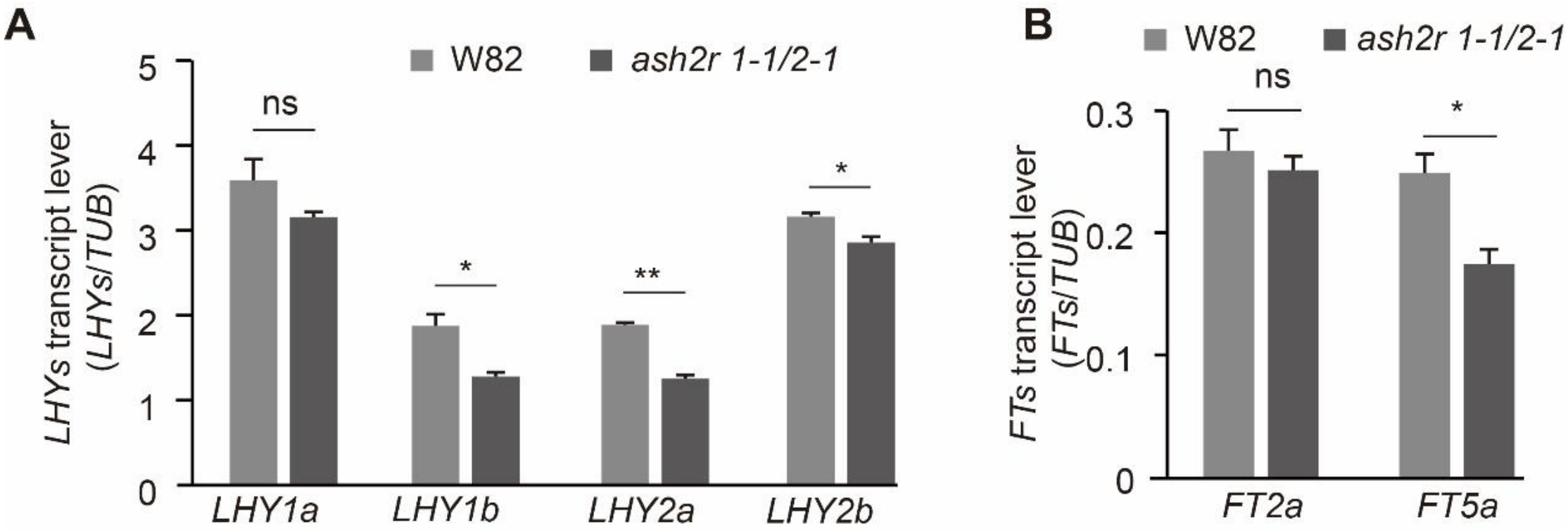

Supplemental Figure 9. Expression analysis of flowering pathway genes in *ash2r1/2* mutants under short-days (SDs). (A-B) Relative transcript levels of *GmLHY1a*, *GmLHY1b*, *GmLHY2a*, *GmLHY2b* **(A)**, and *GmFT2a/5a* **(B)** in WT and *ash2r1-1/2-1* mutants under SDs, quantified by RT-qPCR. All transcript levels were normalized to the endogenous reference gene *Tubulin*. Leaf samples were collected from two-week-old seedlings at ZT4 under SDs. Data represent mean ± s.d. of three biological replicates. Statistical significance was determined by two-tailed Student's t-test: ***P* < 0.01, **P* < 0.05, and ns for not significant.

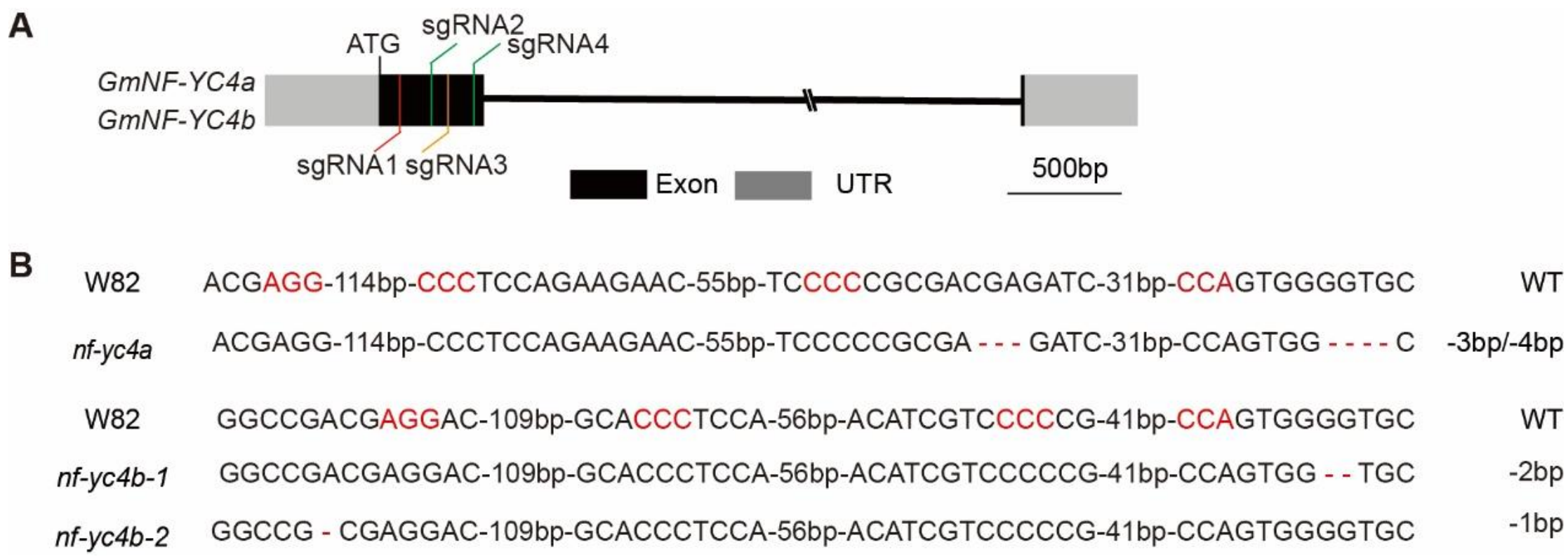

Supplemental Figure 10. Generation and verification of *GmNF-YC4a* and *GmNF-YC4b* mutant lines using CRISPR/Cas9. (A) Schematic representation of sgRNA target sites in *GmNF-YC4a* and *GmNF-YC4b* genomic loci. **(B)** Sequence chromatograms of WT (W82) and mutant alleles. Deleted nucleotides are indicated by orange dashes, with PAM sequences shown in orange font. The sizes of deletions are indicated on the right.

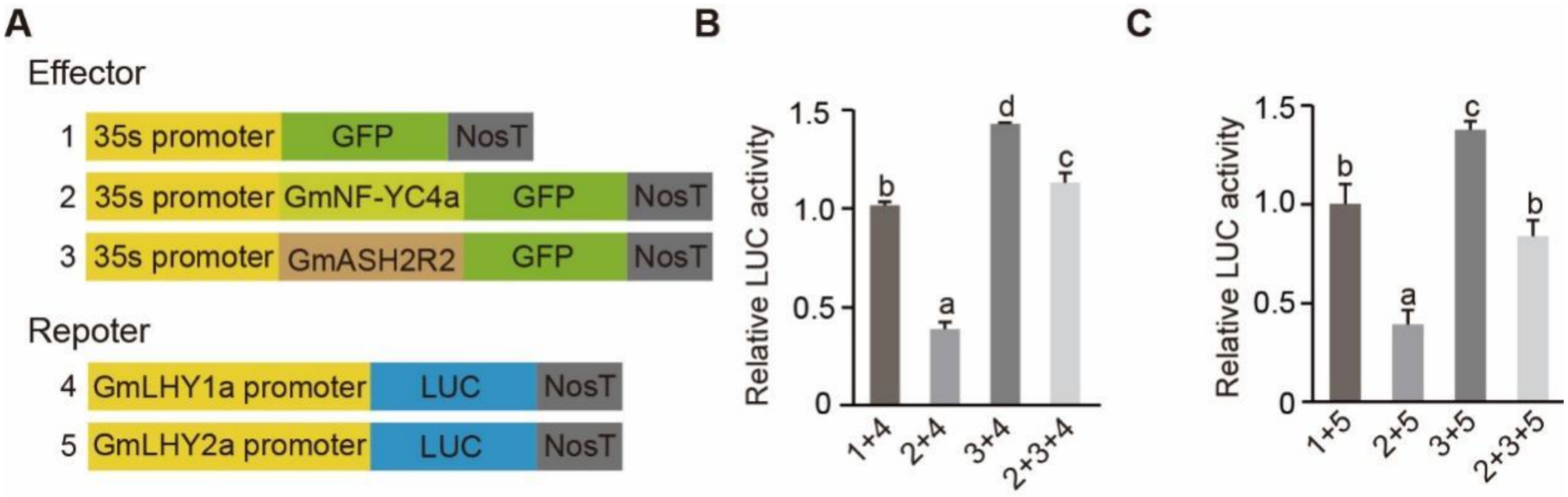

Supplemental Figure 11. GmNF-YC4a and GmASH2R2 antagonistically regulate *GmLHY* promoter activity. (A) Schematic diagram of constructs used for transient expression assays. 35S, CaMV 35S promoter; LUC, firefly luciferase; NosT, nopaline synthase terminator. **(B-C)** Relative luciferase activity driven by the *GmLHY1a* **(B)** and *GmLHY2a* **(C)** promoters when co-expressed with GmNF-YC4a, GmASH2R2, or both proteins in *N. benthamiana* leaves.

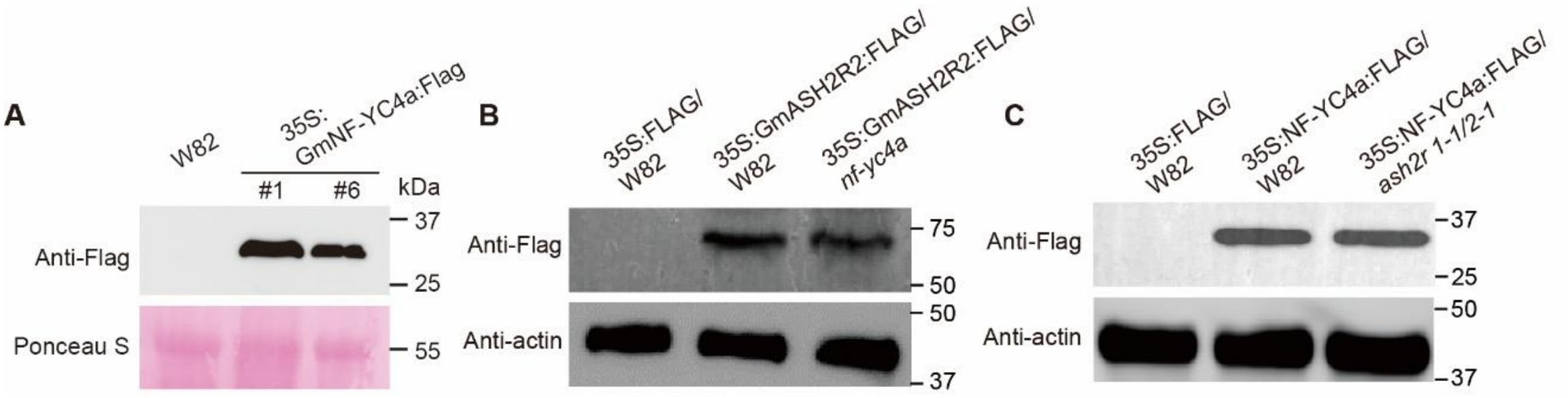

Supplemental Figure 12. Western blot analysis of GmNF-YC4a and GmASH2R2 in soybean overexpression lines and transient transformation assays. (A) Immunoblot analysis of GmNF-YC4a-3×FLAG fusion proteins in transgenic soybean lines. Protein extracts from leaves of overexpression lines grown under LDs were probed with anti-FLAG antibody. Specific bands corresponding to GmNF-YC4-3×FLAG. Ponceau S served as loading control. **(B)** Immunoblot detection of GmASH2R2-3×FLAG fusion proteins in soybean W82 and *nf-yc4a* backgrounds following transient transformation. Total proteins were extracted from leaves at 72 h after transient overexpression. The anti-FLAG antibody was used to detect specific immunoreactive bands corresponding to GmASH2R2-3×FLAG. Soybean Actin was employed as an internal loading control. **(C)** Immunoblot analysis of GmNF-YC4a-3×FLAG fusion proteins in soybean W82 and *ash2r 1-1 2-1* background upon transient transformation. Leaf tissues were harvested 72 h after transient overexpression for total protein extraction. Specific protein bands of the GmNF-YC4a-3×FLAG fusion were immunodetected using an anti-FLAG antibody. Actin served as a reference to ensure equal protein loading across samples.

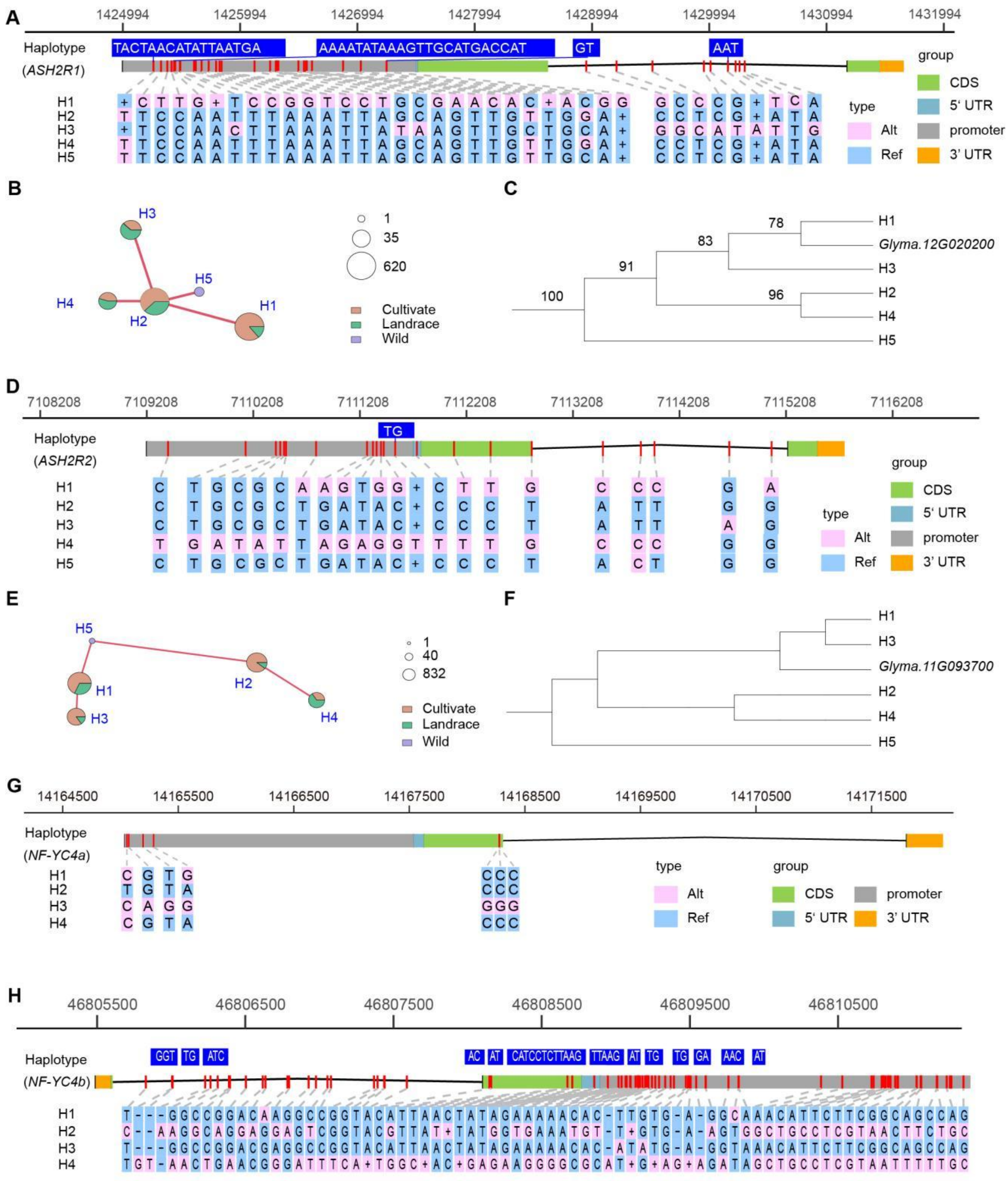

Supplemental Figure 13. Haplotype analysis and evolutionary origins of *GmASH2R1/2* and *GmNF-YC4a/b* in soybean populations. (A, D, G, H) Haplotype structures of *GmASH2R1* **(A)**, *GmASH2R2* **(D)**, *GmNF-YC4a* **(G)**, and *GmNF-YC4b* **(H)**. Gene structures show UTRs (white boxes), coding sequences (dark boxes), and promoter regions (2.5 kb upstream). Nucleotide variants relative to the reference genome are highlighted in red; identical sequences are shaded blue. Scale indicates genomic positions (bp). H1/H2: as described above with Figure 6C. **(B, E)**

Evolutionary origins of *GmASH2R1* **(B)** and *GmASH2R2* **(E)** haplotypes. Pie charts display haplotype distributions among wild (red), landrace (green), and improved cultivar (purple) accessions. **(C, F)** Phylogenetic analysis of *GmASH2R1* **(C)** and *GmASH2R2* **(F)** haplotypes**.** Neighbor-joining trees were constructed using the maximum composite likelihood method with 1,000 bootstrap replicates. Major clades show bootstrap support values.

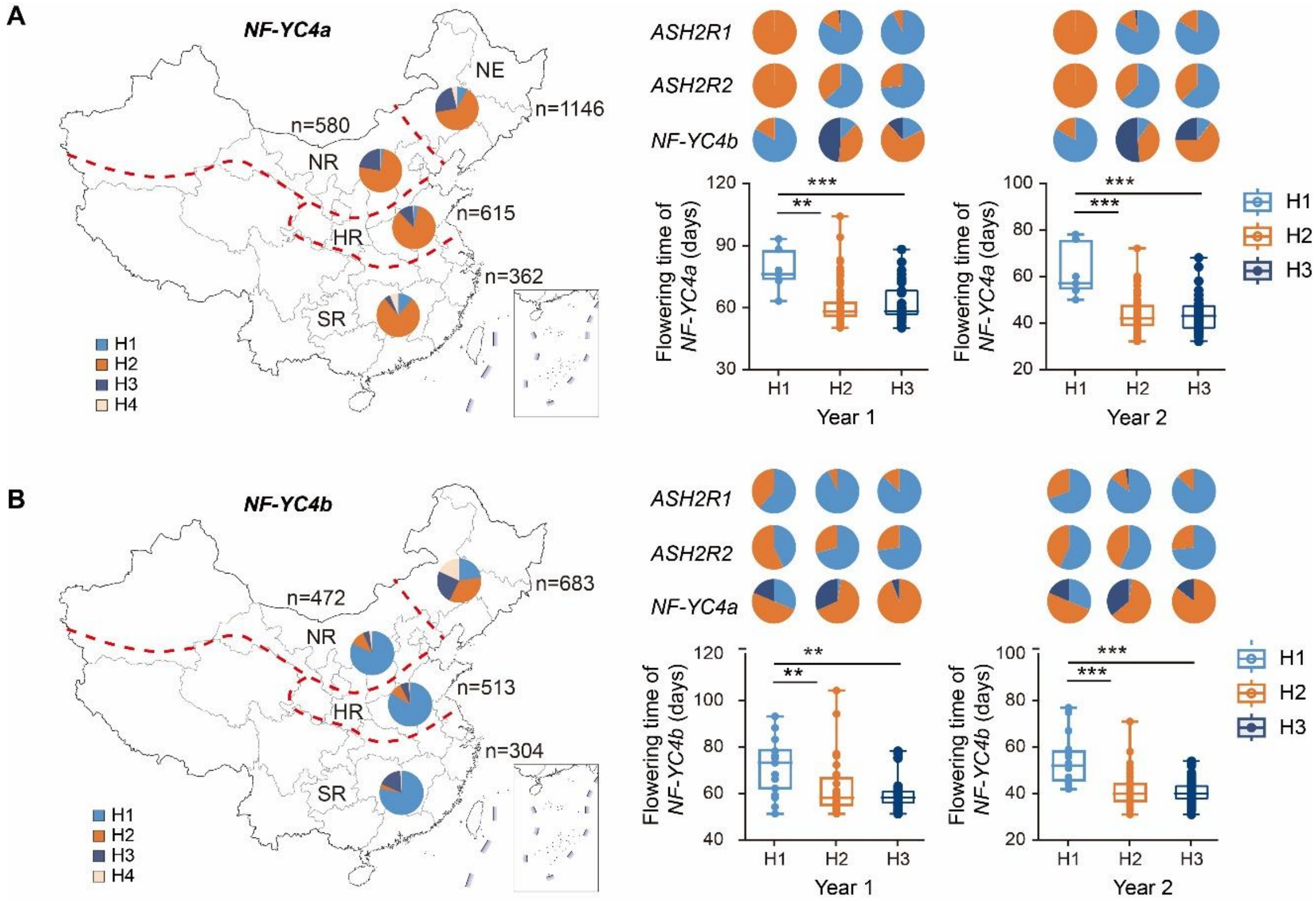

Supplemental Figure 14. Geographic distribution and flowering time associations of *GmNF-YC4a/b* haplotypes link to their antagonistic *GmASH2R1/2* alleles in soybean. (A, B) Spatial distribution and flowering time associations of *GmNF-YC4a* **(A)** and *GmNF-YC4b* **(B)** haplotypes. Maps show the geographical distribution of soybean accessions carrying different haplotypes across four major ecological regions: Northeast China (NE), North China (NR), Huang-huai region (HR), and South China (SR). Pie charts display the relative frequencies of *GmNF-YC4a/b* haplotypes and their antagonistic *GmASH2R1/2* alleles in the analyzed population. Box plots (right) illustrate flowering time variation among accessions with different *GmNF-YC4* haplotypes, based on two-year field trials in Heilongjiang. Boxes span the interquartile range (IQR) with median line; whiskers extend to minimum and maximum values. Statistical significance was determined by two-tailed t-tests: $P < 0.05$, $^{**}P < 0.01$, $^{***}P < 0.001$. H1/H2: as described above with Figure 6C.

Supplementary Table 1: Primers used in this study

Experiment	Amplified region	Sequence (5'to 3' or N to C)
RT-qPCR	*TUB*	F: TCTTGGACAACGAAGCCATCT
		R: GGTGAGGGACGAAATGATCT
	LHY1a	F: GGCCTTTCAGGCTCTGTTCT
		R: AACTGCATTTCTTGCTGCCG
	LHY1b	F: CTGTTCTCCTGCATCGGTGA
		R: ATGGCAGCTGGTGACTTTGA
	LHY2a	F: TGGATCCAGAACACTCAGAAGC
		R: TCGGGGTTCTCAGAAATGACC
	LHY2b	F: AGAACGAGGAGAGATTAAACCACA
		R: GGGTTCTCAGAAATTGCTTGGT
	FT2a	F: TCCCGATGCACCTAGCCCA
		R: ACACCAAACGATGAATCCCCA
	FT5a	F: AGCCCGAACCCTTCAGTAGGGA
		R: GGTGATGACAGTGTCTCTGCCCA
	E1	F: CACTCAAATTAAGCCCTTTCA
		R: TTCATCTCCTCTTCATTTTTGTTG
ChIP-qPCR	*TUB*	F: CCTTCTGTTGTTCCTGGTGGT
		R: CAAACTTGTGGTCAATGCGAG
	LHY1a-P1	F: GCATGACACGAGAATGTGTCAGC
		R: CATTGGCTCGCTGATAAATCTCAG
	LHY1a-P2	F: CGCACCTGATAACAGTGTAACTC
		R: TAAAAGCGTCGCCAGCTTGTGAC
	LHY1a-P3	F: GATTAAAAGCTTAAAGCCTCATCCC
		R: CAATCTCAGCCACTACAATTTCGTG
	LHY1a-P5	F: CTCTTCTTTCGACCAAACAGCTG
		R: TGTAAATTGAGGATGTGCGTTGATAC
	LHY2a-P1	F: AGGATCGAACTTAGGCCTTGAAG
		R: CTTTCAGGTAAGAGTAGGGTATAGC
	LHY2a-P4	F: AAGGTAGCATTGACTGTTCTACATC
		R: AGTGTAGTAATGACCGGCTGTC
	LHY2a-P6	F: ATGGAACGCACAGCAAGTAACG
		R: TCCGACGCCATTGTTTTCAGTC
	LHY2a-P7	F: TTGGCAAGCTTGGGCAATCTG
		R: GCCAGTACTGCTTTGAACTTTGAAG
